# Transcriptome‐wide comparison of selenium hyperaccumulator and nonaccumulator *Stanleya* species provides new insight into key processes mediating the hyperaccumulation syndrome

**DOI:** 10.1111/pbi.12897

**Published:** 2018-03-14

**Authors:** Jiameng Wang, Jennifer J. Cappa, Jonathan P. Harris, Patrick P. Edger, Wen Zhou, J. Chris Pires, Michael Adair, Sarah A. Unruh, Mark P. Simmons, Michela Schiavon, Elizabeth A. H. Pilon‐Smits

**Affiliations:** ^1^ Department of Biology Colorado State University Fort Collins CO USA; ^2^ Department of Horticulture Michigan State University East Lansing MI USA; ^3^ Ecology, Evolutionary Biology and Behavior Michigan State University East Lansing MI USA; ^4^ Department of Statistics Colorado State University Fort Collins CO USA; ^5^ Division of Biological Sciences Bond Life Sciences Center University of Missouri Columbia MO USA; ^6^ DAFNAE University of Padova Padova Italy

**Keywords:** hyperaccumulation, interspecies comparison, RNA‐Seq, selenium, *Stanleya pinnata*, transcriptomics

## Abstract

To obtain better insight into the mechanisms of selenium hyperaccumulation in *Stanleya pinnata*, transcriptome‐wide differences in root and shoot gene expression levels were investigated in *S. pinnata* and related nonaccumulator *Stanleya elata* grown with or without 20 μm selenate. Genes predicted to be involved in sulphate/selenate transport and assimilation or in oxidative stress resistance (glutathione‐related genes and peroxidases) were among the most differentially expressed between species; many showed constitutively elevated expression in *S. pinnata*. A number of defence‐related genes predicted to mediate synthesis and signalling of defence hormones jasmonic acid (JA, reported to induce sulphur assimilatory and glutathione biosynthesis genes), salicylic acid (SA) and ethylene were also more expressed in *S. pinnata* than *S. elata*. Several upstream signalling genes that up‐regulate defence hormone synthesis showed higher expression in *S. pinnata* than *S. elata* and might trigger these selenium‐mediated defence responses. Thus, selenium hyperaccumulation and hypertolerance in *S. pinnata* may be mediated by constitutive, up‐regulated JA, SA and ethylene‐mediated defence systems, associated with elevated expression of genes involved in sulphate/selenate uptake and assimilation or in antioxidant activity. Genes pinpointed in this study may be targets of genetic engineering of plants that may be employed in biofortification or phytoremediation.

## Introduction

Selenium (Se) is chemically similar to sulphur (S), and therefore, selenate (SeO_4_^2−^) can be reductively assimilated in plants and other organisms through the sulphate assimilation pathway (Geering *et al*., 1968). Selenium is essential for many animals, bacteria and certain microalgae, where it has a role in antioxidant and hormone metabolism (Foster and Sumar, 1997). In higher plants, Se is not known to have any essential functions, but it is beneficial at low levels (Pilon‐Smits *et al*., 2009). At elevated Se levels, most plants have stunted growth and chlorosis due to oxidative damage and nonspecific replacement of S with Se in proteins (Ng and Anderson, 1978; Van Hoewyk, 2013).

Some members of Fabaceae (*Astralagus* spp.), Asteraceae (*Symphyotrichum*,* Xylorhiza* and *Oonopsis* spp.) and Brassicaceae (*Stanleya pinnata*) can accumulate Se in their natural habitats to 0.1%–1.5% of their dry weight (DW) and are known as Se hyperaccumulators (Cappa and Pilon‐Smits, 2014; El Mehdawi *et al*., 2014). Such plant Se concentrations are toxic to most animals and fungi, and therefore, hyperaccumulation appears to be a defence strategy against herbivores and pathogens (Freeman *et al*., 2007, 2009; Hanson *et al*., 2004).

Selenium hyperaccumulators could be exploited in phytoremediation programmes aimed to cleanup Se‐contaminated soils, waters and sediments. As an example, different *S. pinnata* genotypes tolerant to salt and boron have been used in a field experiment for Se phytoremediation from agricultural drainage sediments in the San Luis Drain in central California (Freeman and Banuelos, 2011). After two growth seasons, plants and their rhizosphere associated microbes removed about 30% of total Se. Plant material from Se hyperaccumulators grown on naturally seleniferous soils or used in phytoremediation can be also envisioned as soil amendment to enrich food crops in Se‐poor regions of the world (Bañuelos *et al*., 2015; Schiavon and Pilon‐Smits, 2017). In two recent studies, Se‐enriched *S. pinnata*‐derived material was used as soil amendment and proposed as a potential source of organic Se for carrot and broccoli biofortification (Bañuelos *et al*., 2015, 2016).

Alternatively, Se hyperaccumulators may be a source of genes and alleles that can be used for incorporation via genetic engineering into nonaccumulator crop species for Se phytoremediation or biofortification, if the mechanisms by which they tolerate and accumulate Se can be elucidated (Banuelos *et al*., 1997, 2010). In this context, identifying the genes and gene networks responsible for the Se hyperaccumulation syndrome may open the way for the generation of transgenic plants with enhanced Se uptake and/or Se hypertolerance via overexpression of genes encoding transporters with high affinity for Se or rate‐limiting enzymes for Se assimilation/volatilization, as well as via introduction of additional metabolic pathways that may help detoxify Se compounds.

Hyperaccumulators appear to detoxify Se by reducing selenate to less toxic organic forms such as the nonprotein amino acids selenocystathionine and methylselenocysteine (MeSeCys) and sequestering these in peripheral tissues; they also volatilize methylated selenocompounds at high rates, primarily as dimethyl diselenide (DMDSe). Conversely, Se nonaccumulators lack the enzyme responsible for methylation of Se‐cysteine (SeCys) to produce MeSeCys, and their main strategy to reduce Se toxicity in tissues is the conversion of Se‐methionine (SeMet) into the volatile compound dimethyl selenide (DMSe) (Brown and Shrift, 1981; Zayed *et al*., 1998; Meija *et al*., 2002; Freeman *et al*., 2006; Freeman and Banuelos, 2011; Cakir and Ari, 2013). Other detoxification mechanisms may involve enhanced capacity to scavenge free radicals and elevated proteasome activity for recycling of malformed proteins (Freeman *et al*., 2010; Van Hoewyk, 2013).

To obtain insight into Se hyperaccumulation and hypertolerance mechanisms in *S. pinnata*, we investigated transcriptome‐wide differences in gene expression between *S. pinnata* and the Se nonaccumulator *Stanleya elata* (El Mehdawi *et al*., 2012). Phylogenetic and physiological analyses of the *Stanleya* genus showed *S. elata* to be one of four species in a clade that is sister to *S. pinnata* and Se hyperaccumulation to be an apomorphy in the *S. pinnata* species complex (Cappa *et al*., 2015). The two selected species were grown in the presence or absence of selenate and compared with respect to growth, Se and S accumulation and their root and shoot transcriptomes via RNA sequencing. RNA sequencing has become the gold standard for whole‐transcriptome gene expression quantification (Everaert *et al*., 2017). Several transcriptome‐wide studies have validated results from RNA‐Seq with other methods, finding high gene expression correlations (Everaert *et al*., 2017; Liu *et al*., 2011; Marioni *et al*., 2008; Nagalakshmi *et al*., 2008). The full transcriptome approach was chosen because it allows to not only study in more detail the transcript levels in pathways already expected to play a direct role in Se hyperaccumulation and tolerance, but also to discover novel genes and pathways, such as ones that may trigger the signalling cascade events in response to Se.

## Results

### Biomass and Se and S concentrations

No differences in shoot biomass were observed between the treatments when *S. pinnata* and *S. elata* were grown with or without 20 μm selenate (Figure S1A). Similarly, in an identical experiment carried out for elemental analysis, there were no differences in biomass production between treatments (Figure S1B). Moreover, the Se level chosen did not cause visible symptoms of toxicity. *S. pinnata* had a higher shoot Se concentration than *S. elata*, both when supplied with selenate and without Se (background Se from seed), and both species had higher Se levels when treated with Se than without (Figure S1C). The shoot sulphur (S) concentration was higher in *S. pinnata* than *S. elata* when treated with Se; in the absence of Se, no difference was observed between the species (Figure S1D). Within *S. elata*, Se treatment did not affect S levels, whereas in *S. pinnata,* S levels were higher in the presence of Se (Figure S1D).

### Transcriptome analysis overview

We assembled *de novo* 63 859 089 bp of *S. pinnata* sequence and 64 685 441 bp of *S. elata* sequence. Contigs shorter than 300 bp were removed; the average contig length was 594 bp. A total of 105 280 contigs (longer than 300 bp) were assembled for *S. pinnata* and 101 616 contigs for *S. elata*. Approximately 93% of these contigs were successfully annotated to *A. thaliana*. The ATID numbers to which the BLASTed contigs were used as IDs for the corresponding *Stanleya* genes, and the sum of RPKMs was used as a measure for gene expression. 19 572 genes were matched in both *Stanleya* species. After geometric means adjustment, 443 genes were removed due to negligible expression and/or within‐sample variations. The remaining 19 129 genes were used for all statistical analysis thereafter. In addition to expression comparisons within species, the expression levels of *S. pinnata* and *S. elata* genes associated with the same ATID were statistically compared to each other. These analyses included Se effect, species effect, organ effect and their interactions, as described in the methods (including some text in Appendix S1).

**Figure 1:**
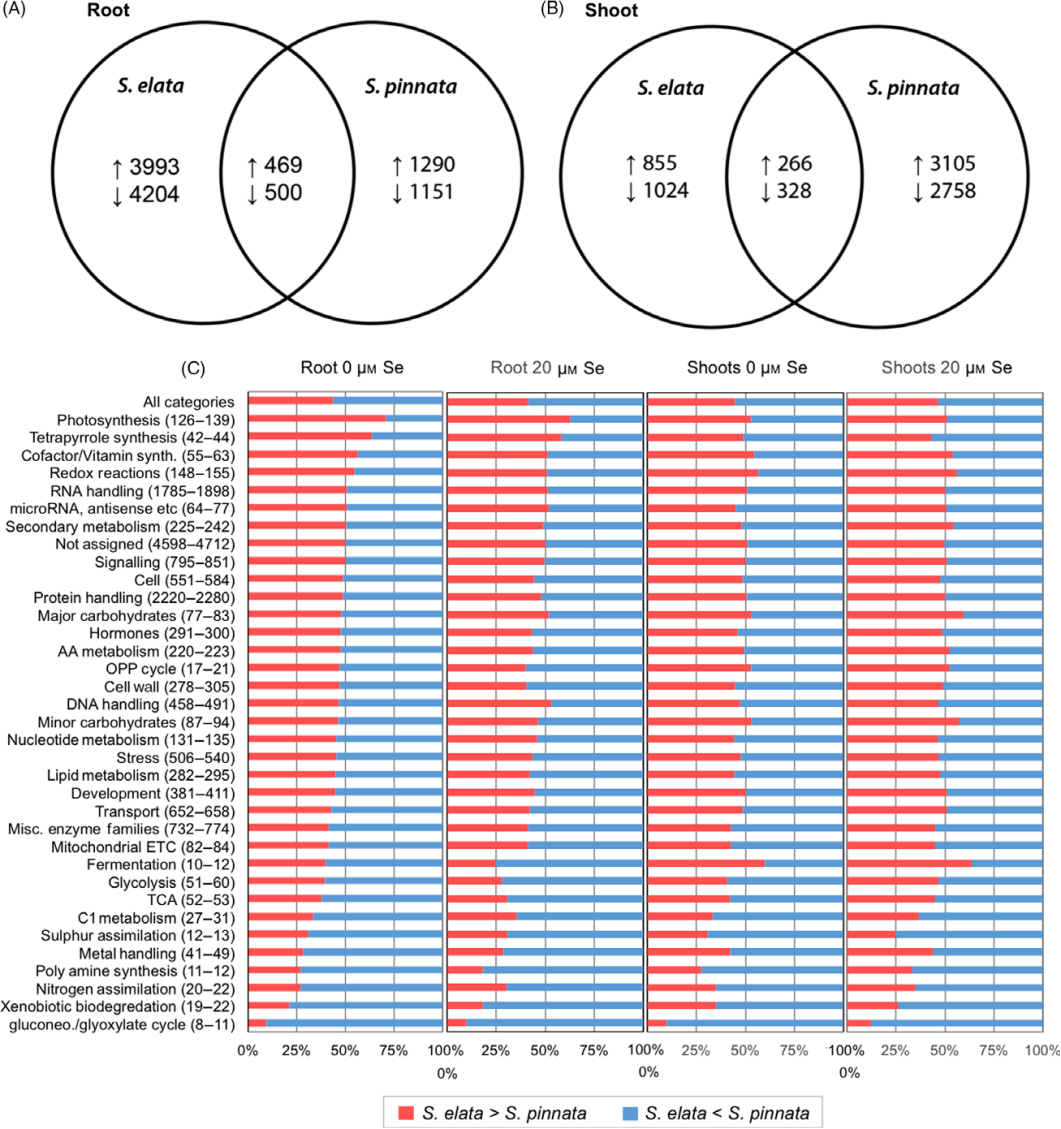
Venn diagrams showing the numbers of transcripts in *Stanleya pinnata* or *S. elata* that were significantly increased (↑) or decreased (↓) in expression by 20 μm Se in roots (A) and shoots (B). Overlapping areas represent genes with shared regulation patterns between species. (C) Differential expression patterns between *S. pinnata* and *S. elata* for major functional categories, as sorted by Mapman. The bracketed numbers to the right of the category names indicate the range in the number of genes identified for that category for all four treatments: roots or shoots, 0 or 20 μm Se. For each treatment, red bars signify the percentage of genes with higher expression in *S. elata* than *S. pinnata* for a category, while blue bars signify the percentage of genes with higher expression in *S. pinnata* than *S. elata* (*q *<* *0.005).

An overview of gene expression responses to Se treatment is shown in Figure 1. In roots, many more genes responded to Se treatment (*q* < 0.005) in *S. elata* compared to *S. pinnata* (Figure 1A), while in shoots, more genes were affected by Se treatment in *S. pinnata* than in *S. elata* (Figure 1B). The transcript levels of ~1000 genes were similarly affected by Se in both plant species for roots, versus ~600 for shoots; both were relatively small fractions compared to genes differently affected by Se treatment in the two species. Gene expression differences between *S. pinnata* and *S. elata* were analysed by comparing RPKM values of genes annotated to the same *A. thaliana* locus (Figure S2). In both roots and shoots, the majority of genes were differentially expressed between the two *Stanleya* species, and a large proportion of these genes were differentially expressed in both –Se and +Se treatments. Approximately half of the differentially expressed genes were expressed more in *S. pinnata* than *S. elata*, and the other half more in *S. elata* than *S. pinnata*. Among genes that were differentially expressed between species under only one treatment, the numbers of genes were similar for the +Se and −Se treatments; this was true for both organs (Figure S2).

As a first approach to identify gene families that may be particularly important for Se accumulation or tolerance in *S. pinnata*, we listed the top 100 genes most affected by Se treatment in each species and organ (Table S1), as well as the top 100 genes most differentially expressed between the two plant species for different Se treatments and organs (Table S2). This number of 100 was chosen arbitrarily. Some gene families prominently present in these lists include antioxidant‐related genes (particularly peroxidases were expressed more highly in *S. pinnata* than *S. elata*), defence‐related genes (particularly major latex proteins (*Mlp*s), for which expression was dependent on both Se and *Stanleya* species), sulphate assimilation genes (particularly adenosine triphosphate (ATP) sulphurylases and adenosine‐phosphosulphate (APS) reductases, which were expressed at higher levels in *S. pinnata*), transcription factors (particularly zinc finger protein genes, which differed with both Se treatment and *Stanleya* species), as well as glutathione‐S‐transferases (GSTs) and methyltransferases (particularly S‐adenosyl methionine‐dependent methyltransferases).

The genes that showed differential expression between the two species were mapped into functional groups through Mapman (see methods), to observe whether certain groups as a whole were more expressed in hyperaccumulator or nonaccumulator. Across all functional groups combined (Figure 1C, top bar), slightly more than half (55%) of the differentially expressed genes were more highly expressed in *S. pinnata* than *S. elata*. Notable functional groups in that were overall more highly expressed in the Se hyperaccumulator, regardless of organ and Se treatment, included xenobiotic biodegradation, nitrogen assimilation and polyamine synthesis, metal transport/detoxification and S assimilation. We did not identify any functional groups of genes that were more highly expressed in *S. elata* across all treatments.

The Mapman bin denoted ‘metabolic pathways and large protein families’ was analysed in Mapman for +Se and –Se, in both roots and shoots, to gain insight into which specific functional groups of genes within this category were most differentially expressed between the two plant species. Genes that were associated with peroxidases had significantly (*P* < 0.05) deviated expression compared with other gene groups displayed in the pathway for +Se and –Se in roots. The peroxidase group also had a significantly higher proportion of genes with greater expression in *S. pinnata* relative to other gene groups, as the *P*‐value was < 0.05 or the mean average deviation (MAD) score was larger than 95th quantile for the majority of treatments. Additionally, glutathione‐S‐transferase genes had significantly different expression in roots treated with Se treatment (*P* < 0.05) compared with other gene groups, as well as a higher proportion (*P* < 0.05) of genes with greater expression in *S. pinnata* compared to other gene groups. No gene groups were found to deviate in expression from other gene groups in shoots for +Se or –Se treatments.

The next sections highlight the transcriptome results from selected functional groups of genes that stood out in this and earlier studies. The focus is on species differences, as those were of primary interest in this investigation. To denote the different *Stanleya* transcripts, the *A. thaliana* gene name and number is used that was found to have the highest sequence similarity.

### Transcript analysis of genes involved in sulphate/selenate transport and assimilation

**Figure 2:**
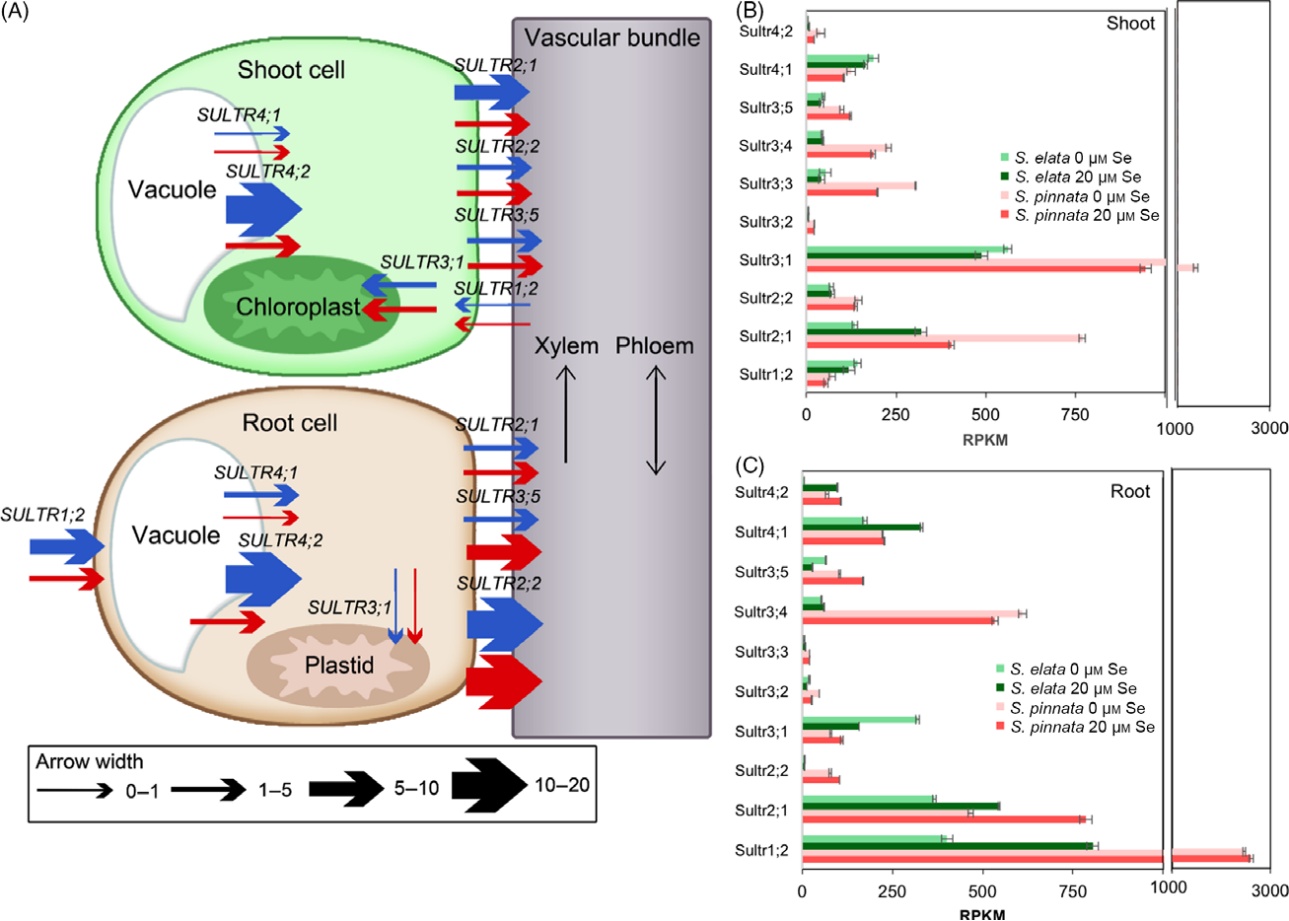
Expression levels (TMM‐normalized RPKM values) of sulphate transporter (*Sultr*) genes in shoots and roots of *Stanleya pinnata* and *S. elata* grown on 0 or 20 μm sodium selenate. (A) Schematic representation of the differences in expression levels between *S. pinnata* and *S. elata* for SULTR genes. Width of arrows represents the fold difference between species (ratio of *S. pinnata* RPKM/*S. elata* RPKM) for a given treatment and organ. Blue arrows are for plants grown without Se and red arrows for plants grown with 20 μm Se. (B) Shoot and (C) root expression levels of *Sultr* genes (*n* = 3, mean ± SD). Significant differences between treatments are presented in the text.

The transcript levels in the sulphate transporter (*sultr)* gene family are of particular interest as they mediate sulphate/selenate uptake and translocation. The arrow thickness in Figure 2A shows the fold differences (based on the normalized RPKM values shown in the same figure) in *sultr* expression levels between *S. pinnata* and *S. elata*, both in the presence and absence of Se, and depicts their roles in sulphate/selenate transport. Figure 2B,C shows the transcript levels of *sultr* genes differentially expressed between the plant species. *S. pinnata* had higher transcript levels compared to *S. elata* for several important *sultr* genes, including *sultr1;2* (root uptake), *sultr2;1* and *sultr3;5* (root‐to‐shoot translocation), *sultr4;2* (vacuolar efflux, also contributing to translocation), *sultr2;2* (phloem remobilization), *sultr3;1* (plastid import), *sultr3;3* and *sultr3;4* (function unknown, Kataoka *et al*., 2004; Takahashi *et al*., 2011; Cao *et al*., 2013). In an independent experiment, 10‐fold elevated transcript levels of *sultr1;2* in *S. pinnata* relative to *S. elata* were also observed when plants were grown in hydroponics in the absence of Se (Figure S3).

**Figure 3:**
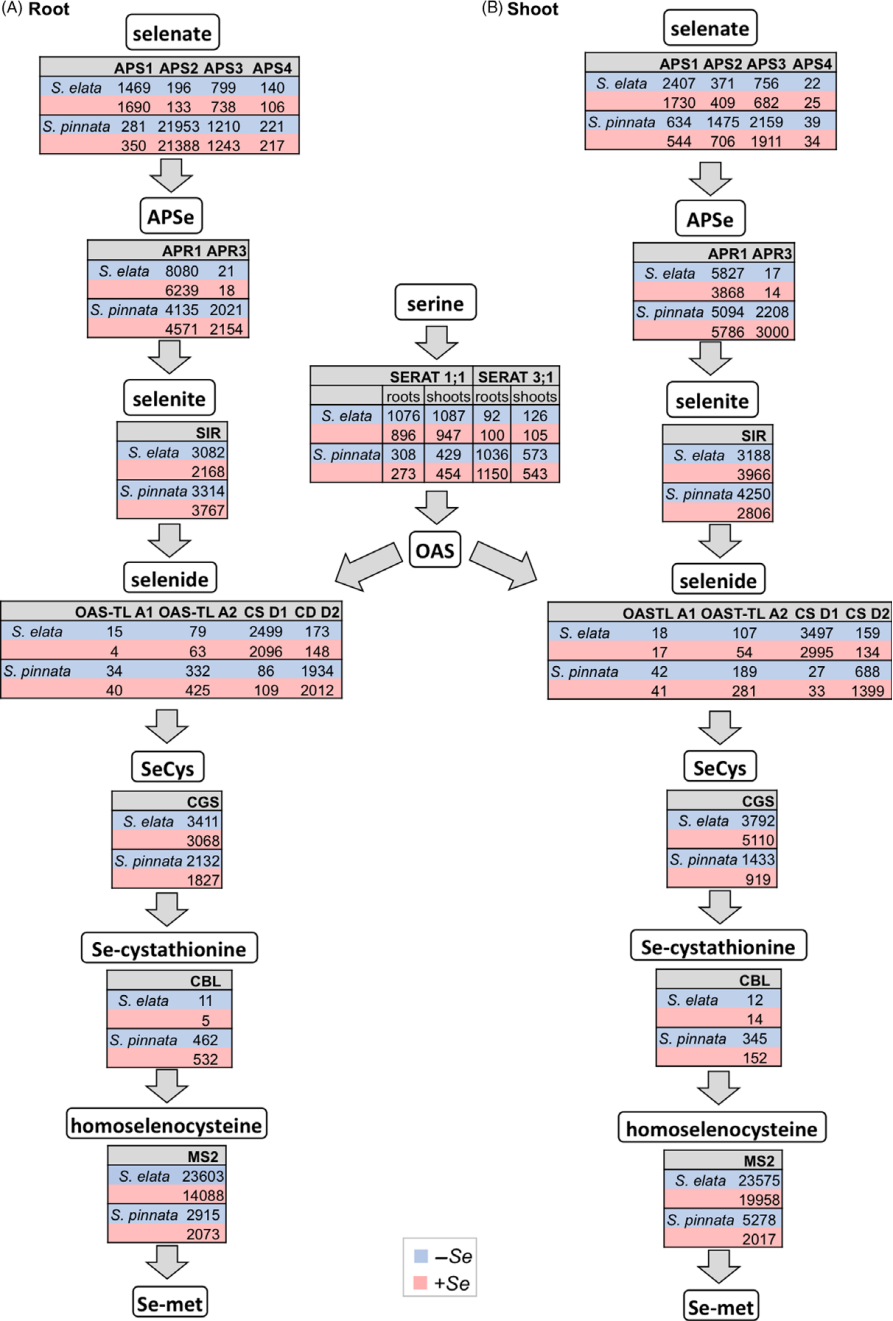
Expression levels (TMM‐normalized RPKM values) of genes involved in Se/S assimilation in roots (A) and shoots (B) of *Stanleya pinnata* and *S. elata* grown on 0 or 20 μm sodium selenate. Values displayed are the mean of three bioreplicates per treatment.

As shown in the flow diagram in Figure 3A,B, the transcript levels of sulphate assimilation genes differed greatly in the two plant species. Key enzyme ATP sulphurylase 2 (*aps2*) showed extremely high transcript levels in *S. pinnata* roots, over 120‐fold higher compared to *S. elata*, both for –Se and +Se treatments. In shoots, *aps2* expression was twofold to fourfold higher in *S. pinnata* than *S. elata*. Among the other *aps* genes, *aps3* and *aps4* transcript levels also were higher in *S. pinnata* than *S. elata*, but *aps1* showed higher expression in *S. elata* compared to *S. pinnata*. In an independent experiment where plants were grown in hydroponics in the absence of Se, elevated transcript levels of *aps2* in *S. pinnata* relative to *S. elata* were also observed; in the same hydroponic experiment, the *aps1* transcript levels were somewhat lower in *S. pinnata* than *S. elata*, in agreement with the transcriptome data (Figure S3).

Among the APS reductase (*apr*) genes, *apr3* was expressed at ~100‐fold higher level in *S. pinnata* than *S. elata; apr1* was expressed in *S. elata* roots at ~1.5‐ to twofold higher levels compared to *S. pinnata* roots, but in shoots the expression was similar. Furthermore, the expression of APS kinase 1 (*apk1*) was twofold to threefold higher in *S. pinnata* than *S. elata*. This cytosolic enzyme phosphorylates APS to phospho‐adenosine phosphosulphate (PAPS), forming a starting point for the synthesis of a variety of sulphated metabolites including glucosinolates as well as phospho‐adenosine phosphate (PAP), a signal molecule that up‐regulates abiotic stress resistance genes (Bohrer *et al*., 2015; Bruggeman *et al*., 2016).

Among genes encoding serine acetyltransferases (*serat*), which provide O‐acetylserine (OAS) to be combined with sulphide for cysteine (Cys) production (Kawashima *et al*., 2005), *serat1;1* transcripts were more abundant (twofold to threefold) in *S. elata* compared to *S. pinnata*, whereas *serat3; 1* showed much higher expression (fivefold to 10‐fold) in *S. pinnata* than *S. elata*. Among genes directly involved in Cys production, O‐acetylserine thiol lyase (*oas‐tl)A1, oas‐tlA2* and cysteine synthase (*cs)D2* were expressed at higher levels in *S. pinnata* than *S. elata*, whereas *S. elata* had higher expression of *csD1* (Figure 3).

Among the genes involved in metabolism of Cys to methionine (Met), cystathionine gamma synthase (*cgs*) showed similar high transcript levels across the species in the root, while in shoots of plants grown with Se *S. elata* had ~5‐fold greater *cgs* expression than *S. pinnata* (Figure 3). Cystathionine beta lyase (*cbl*) had much higher expression (up to 100‐fold) in *S. pinnata* relative to *S. elata*. Methionine synthase (*ms2*) was expressed in *S. elata* roots and shoots at extremely high levels, fivefold to 10‐fold greater than in *S. pinnata* (Figure 3).

As *S. pinnata* accumulates Se mainly in the form of methyl‐SeCys, it is interesting to note that there was a striking difference (500‐fold to 2000‐fold) between *S. pinnata* and *S. elata* in the transcript levels of a homologue of AT5G40780, which encodes lysine histidine transporter 1 (LHT1), a high‐affinity transporter for cellular uptake of a broad range of amino acids (lysine, histidine, proline, cysteine and others) into cells, including root epidermis and leaf mesophyll (Hirner *et al*., 2006). This difference was especially pronounced in roots of Se‐treated plants. There were no conspicuous differences in expression levels of other predicted amino acid transporters.

### Transcript analysis of genes involved in antioxidant systems

**Figure 4:**
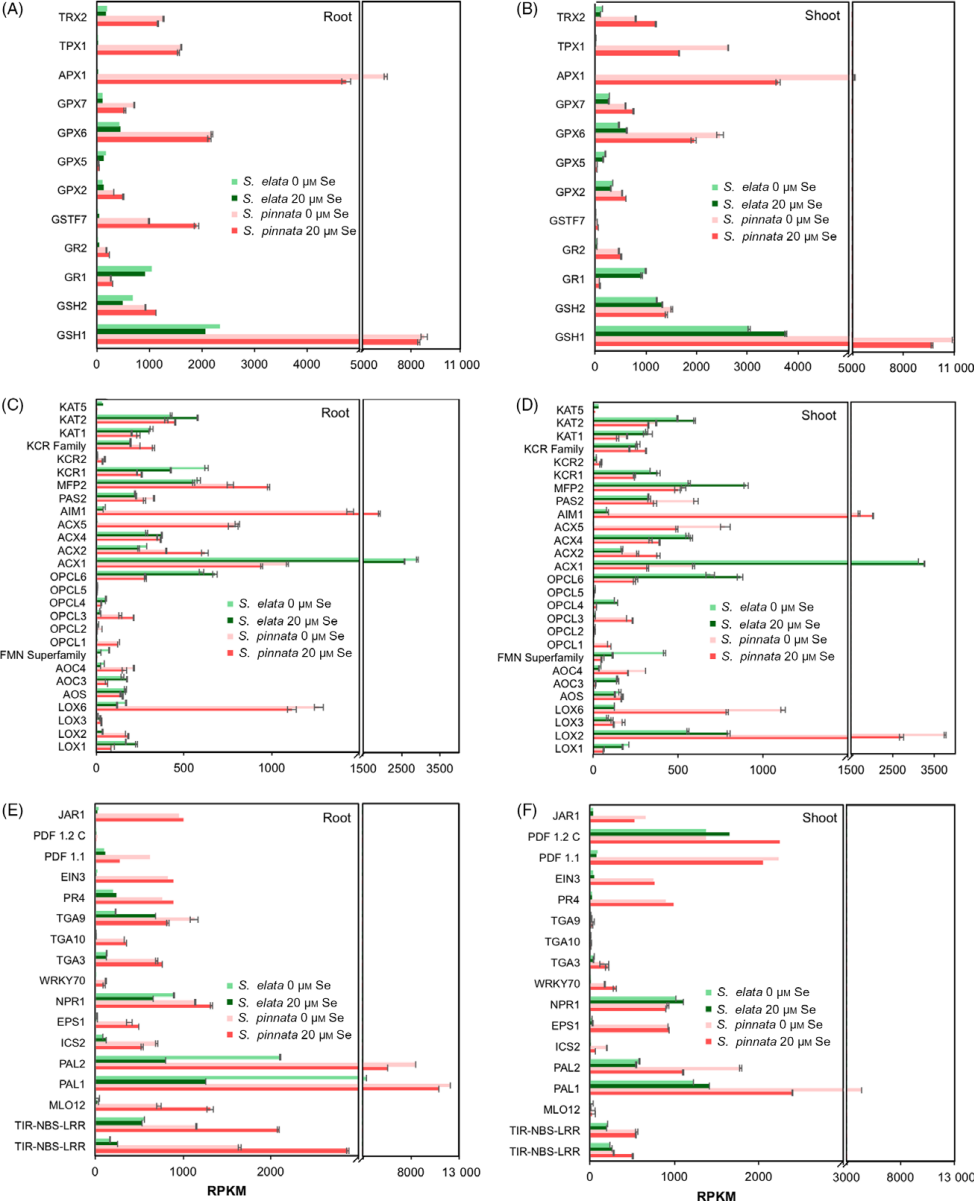
Expression levels (TMM‐normalized RPKM values) of genes involved in antioxidant functions in roots (A) and shoots (B) of *Stanleya pinnata* and *S. elata* grown on 0 or 20 μm sodium selenate and expression levels of genes involved in synthesis and signalling of JA, ethylene and SA that were differentially expressed in roots (C, E) and shoots (D, F) of *S. pinnata* and *S. elata* grown on 0 or 20 μm sodium selenate. Values shown represent the mean (*n* = 3 bioreplicates) ± SD. (C, D) Genes involved in JA biosynthesis. (E, F) Genes involved in ethylene and SA biosynthesis, in JA/SA/ethylene signalling and defence. GSH1: gamma‐glutamylcysteine synthetase; GSH2: glutathione synthetase; GR: glutathione reductase; GSTF: glutathione‐S‐transferase; GPX: glutathione peroxidase; APX: ascorbate peroxidase; TPX: thioredoxin peroxidase; TRX: thioredoxin reductase. MLO: mildew resistance locus O; PAL: phenylalanine ammonia‐lyase; ICS: isochorismate synthase; EPS: enhanced *Pseudomonas* susceptibility; NPR: nonexpressed pathogen resistance genes; WRKY: transcription factor; TGA: TGACG‐binding protein; PR: pathogen resistant; EIN: ethylene insensitive; PDF: pathogen defensin factor; and JAR: jasmonate‐responsive.

Among the genes involved in the biosynthesis of glutathione (GSH) from Cys, Glu and Gly, *gsh1* had extremely high expression in *S. pinnata*, which was fourfold higher than in *S. elata*;* gsh2* transcript levels were marginally higher in *S. pinnata* (Figure 4A,B). GSH reductase *gr1* was more highly expressed in *S. elata* whereas *gr2* was more highly expressed in *S. pinnata* (Figure 4A,B). In the glutathione‐S‐transferase family, which conjugates GSH to various organic substrates, *gstf7* was 30‐fold to 40‐fold more expressed in the roots in *S. pinnata* than *S. elata* (Figure 4A). Transcript levels of several antioxidant enzymes involved in ROS scavenging were much higher in *S. pinnata* than *S. elata* (Figure 4A,B): GSH peroxidases (*gpx2*,* gpx6* and *gpx7*), ascorbate peroxidase (*apx1*)*,* thioredoxin peroxidase (*tpx*) and thioredoxin reductase (*trx2*); only *gpx5* expression was lower in *S. pinnata* (Figure 4A,B).

Another class of genes that may help plants withstand the oxidative stress associated with Se are those implied in the repair, refolding or recycling of damaged proteins. Indeed, the transcript levels of several heat shock proteins (hsp) and chaperones (*hsp70, hsp81.4, hsp89.1, tcp‐1*) were 10‐fold to 30‐fold higher in *S. pinnata* than *S. elata*, as were several ubiquitin‐related genes (*ubc10, rpn1B, ubp23, paa200*).

### Transcript analysis of genes involved in JA, SA and ethylene signalling

Among genes involved in JA synthesis (Figure 4C,D), those at least fivefold more expressed in *S. pinnata* than *S. elata* included *lox2* and *lox6* (lipoxygenase, the first enzyme in JA synthesis), *acx5* and *aim1* (both acyl‐CoA oxidases) and OPC‐CoA ligase (*opcl)1* (At1 g20480) and *opcl3* (At1 g20500). Genes expressed at higher levels (up to sixfold) in *S. elata* than *S. pinnata* include *acx1* (acyl‐CoA oxidase) and *opcl6*.

Figure 4E,F shows the expression of genes involved in SA and ethylene biosynthesis as well as in JA, SA and ethylene signalling and defence responses. Many of these genes had higher expression in the Se hyperaccumulator. These include three genes involved in SA biosynthesis: *pal1*,* pal2* encoding for phenylalanine ammonia‐lyase, the first enzyme of the phenylpropanoids pathway (Chen *et al*., 2009; Dempsey *et al*., 2011), and isochorismate synthase (*ics2*). The gene encoding ethylene insensitive (EIN)3, which responds to ethylene and functions as a transcription factor for downstream processes (Chao *et al*., 1997), showed 10‐fold to 20‐fold higher expression in *S. pinnata*. *Jar1*, a gene involved in JA activation and signalling (Laurie‐Berry *et al*., 2006), was expressed at 10‐fold to 20‐fold higher levels in *S. pinnata* than *S. elata*. Genes involved in SA signal transduction that were more expressed in *S. pinnata* than *S. elata* include *tga3*,* tga9* and *tga10,* which are known to activate the expression of pathogenesis‐related (PR) proteins (Johnson *et al*., 2003). Indeed, *pr4* transcript was 4 to 20 times more abundant in the Se hyperaccumulator (Figure 4E,F). Transcription factor *wrky70*, thought to activate SA‐induced genes (Li *et al*., 2004) was also expressed at higher level in *S. pinnata*. The plant defensin factors (PDF) *pdf1.1* and (to a lesser extent) *pdf1.2c* were also expressed at higher levels in *S. pinnata* than *S. elata* (Figure 4E,F).

As genes involved in plant defence mechanisms were expressed at higher levels in the Se hyperaccumulator, one might expect that there is an up‐regulated upstream receptor triggering this response. Further analysis of the transcriptome in search for possible candidates revealed several genes reported to trigger defence responses that were significantly more expressed in *S. pinnata* than *S. elata*, particularly in roots and in the presence of Se. A homologue of *mlo12* (mildew resistance locus), a plasma membrane protein involved in fungal resistance (Buschges *et al*., 1997), was present at up to 70‐fold higher levels in *S. pinnata* (Figure 4E,F). Also overexpressed in *S. pinnata* were several homologues of nucleotide‐binding site–leucine‐rich repeat (LRR) membrane proteins (Figure 4E,F) involved in pathogen sensing (TAIR).

## Discussion

**Figure 5:**
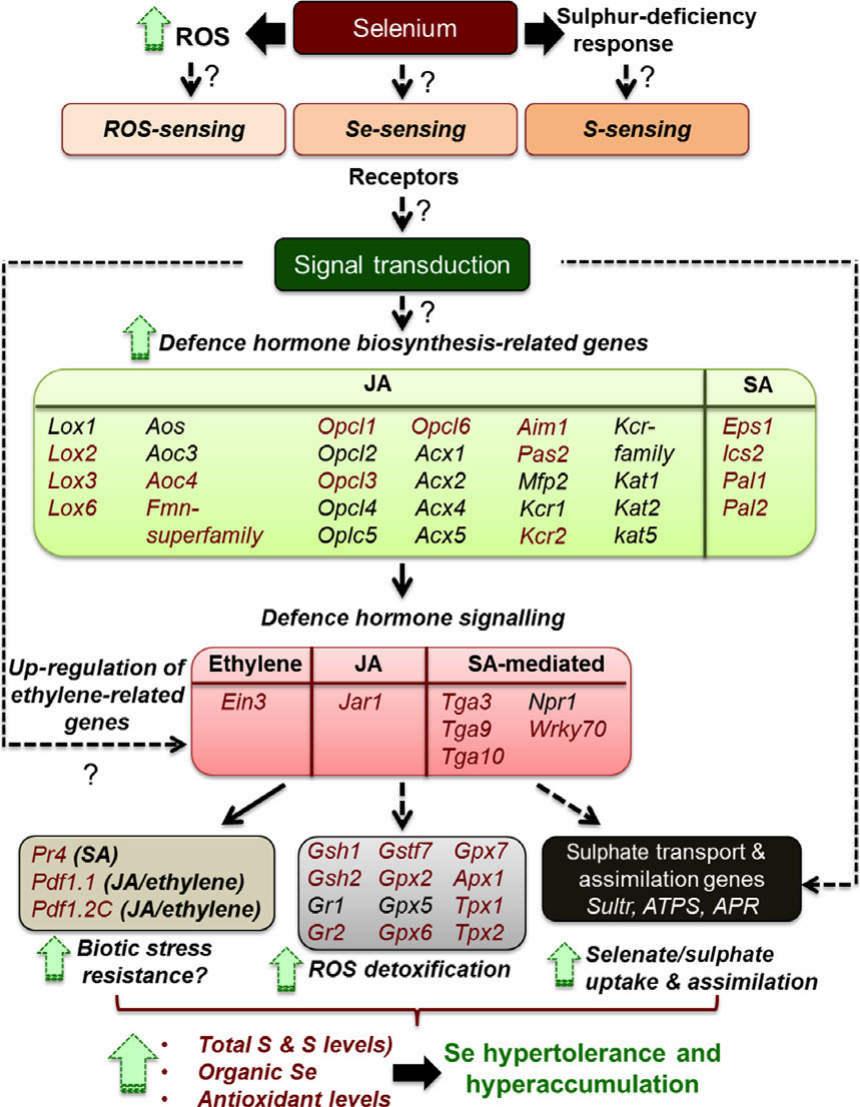
Schematic model of genes proposed to mediate Se hyperaccumulation and hypertolerance in *Stanleya pinnata* based on this transcriptome study. The defence pathways are up‐regulated, leading to increased JA, SA and ethylene hormone synthesis and an increase in overall ROS scavenging ability and S/Se accumulation. Genes in red font were found in this study to be more highly expressed in *S. pinnata* than *S. elata* for all treatments; genes in black font were more expressed in some of the treatments. Solid arrows connecting gene groups represent well‐known interactions established by previous literature; dashed arrows represent tentative connections based on a few previous studies; dashed arrows with question marks represent relationships proposed in this study, suggested for further analysis.

Based on transcriptome comparison of *S. pinnata* with *S. elata*, the Se hyperaccumulator appears to use multiple mechanisms to hyperaccumulate Se and be hypertolerant to Se. A summarizing model is presented in Figure 5. Based on this transcriptome comparison it may be hypothesized that the hyperaccumulator has increased uptake and translocation of selenate through *sultr* transporters, increased assimilation of selenate into organic forms and increased antioxidant and protein repair/recycling capacity, compared to the nonaccumulator. The model further hypothesizes that these processes are regulated via elevated levels of the hormones JA, ethylene and SA, and that their synthesis is up‐regulated in response to elevated levels of certain defence‐related receptors and transcription factors. Defence pathways also appear to be up‐regulated in the hyperaccumulator, likely triggered by these same hormones.

The constitutively elevated expression of *sultr1;2* in *S. pinnata* supports the hypothesis that enhanced *sultr1;2* transport activity is an important mechanism in the hyperaccumulation of Se. In *A. thaliana*,* sultr1;2* is also the main portal for selenate into the plant (El Kassis *et al*., 2007). The expression of *sultr2;1*,* sultr2;2* and *sultr3;5*, which are thought to mediate the flux of sulphate through phloem and xylem parenchyma cells (Kataoka *et al*., 2004), were also higher in *S. pinnata* than *S. elata*. This explains why *S. pinnata* had a greater rate of Se root‐to‐shoot translocation and source‐to‐sink remobilization than *S. elata* in an earlier study (Cappa *et al*., 2014). In hyperaccumulator *Astragalus* species, groups 1 through 4 *sultr* genes were also found to be constitutively up‐regulated (Cabannes *et al*., 2011).

The higher transcript levels of S/Se assimilation genes in *S. pinnata* are in agreement with earlier findings from a ~350‐gene macroarray study (Freeman *et al*., 2010) and explain the finding that *S. pinnata* accumulated no detectable inorganic Se, whereas *S. elata* contained 20%–25% inorganic Se (Cappa *et al*., 2015). More efficient conversion from inorganic to organic Se likely is one of the Se hypertolerance mechanisms in *S. pinnata*.

The two plant species differed in their apparent predominant isoforms for APS, APR, SERAT and cysteine synthase, which may have different kinetic and substrate affinity properties as well as different cellular localization. Isoform *aps2*, for instance, which was particularly up‐regulated in *S. pinnata*, was shown in *A. thaliana* to be dual‐localized to the cytosol and plastids, and constitutes the only cytosolic APS activity (Bohrer *et al*., 2015). The high expression of *aps2* is also significant in view of the finding that APS was found to be a rate‐limiting enzyme for selenate assimilation to organic Se in *Brassica juncea* (Pilon‐Smits *et al*., 2009). It is intriguing that there was extremely high expression of *aps2* in the roots of *S. pinnata*, which may indicate that selenate is assimilated in part in the roots, and that part of the Se may be transported in organic form in the xylem. Indeed, substantial quantities of organic Se, present as seleno‐amino acids, have been detected in the roots of *S. pinnata* (Lindblom *et al*., 2013) as well as in guttation (xylem) fluid (Freeman *et al*., 2006). It is still unclear how these organic seleno‐amino acids are transported across membranes in *S. pinnata*; the homologue of *lht1* (high‐affinity broad substrate amino acid transporter) that was 500‐fold to 2000‐fold more highly expressed in the hyperaccumulator will be an interesting candidate to further investigate.

APS reductase (APR) has also been reported to be a rate‐limiting enzyme for S/Se assimilation (Bick and Leustek, 1998). While *apr3* expression levels were 100‐fold higher in *S. pinnata*,* apr1* expression was twofold lower than in *S. elata* (Figure 5) and both isoforms are plastidic, so whether these expression differences have a physiological consequence is hard to predict. Selenite reduction may be mediated by GSH, either with or without the involvement of GR (Hsieh and Ganther, 1975; Sors *et al*., 2005). The apparent up‐regulation of the S assimilation pathway in *S. pinnata* may give rise to higher GSH levels, as was found in transgenic *B. juncea* overexpressing APS (Pilon‐Smits *et al*., 2009). In addition, genes involved in GSH synthesis (*gsh1*) and reduction (*gr2*) were expressed at higher levels in *S. pinnata* than *S. elata* (Figure 5).

Serine acetyltransferases are generally regulated by feedback inhibition from Cys, whose synthesis depends on combined *serat* and *Oas‐tl* activity (Kawashima *et al*., 2005). In this context, it is interesting that *S. pinnata* had higher root expression levels than *S. elata* of *serat3;1*, which was reported to be insensitive to Cys levels (Kawashima *et al*., 2005). *Stanleya elata* had higher expression of *serat1;1*, which was reported to be sensitive to Cys (Krueger *et al*., 2009). Thus, SeCys synthesis may be more inhibited by Cys accumulation in *S. elata* than *S. pinnata*. Among the *cs* isoforms, the transcript of *cs D2* (mitochondrial and cytosolic) was much more abundant in *S. pinnata* whereas *cs D1* (cytosolic) was much more abundant in *S. elata*. Cytosolic *cs* was reported to negatively regulate root *sultr1;2* activity by binding to its C‐terminal STAS domain (Shibagaki and Grossman, 2010), so this differential expression of *cs* between the species could affect the rate of selenate uptake in *S. pinnata* versus *S. elata*. It is also interesting to note that mitochondrial *cs* activity (e.g. *cs* D2) was shown to be the most important *cs* for Cys synthesis in *A. thaliana* (Birke *et al*., 2012).

Transcript‐level differences for genes mediating the conversion of (Se) Cys to (Se)Met, that is cystathionine‐γ‐synthase (*cgs),* cystathionine‐β‐lyase (*cbl)* and methionine synthase *(ms*) indicate further differences in metabolic Se fluxes in *S. pinnata* and *S. elata*. While *cgs* and *ms2 s*howed higher expression in *S. elata*,* cbl* was more highly expressed in *S. pinnata*. The finding that *cgs* expression was higher than *cbl* expression may explain why *S. pinnata* accumulates around 12% of its Se as selenocystathionine (Freeman *et al*., 2006). The difference between *cgs* and *cbl* transcript levels was even higher in *S. elata*, which may indicate that *S. elata* accumulates a larger fraction of its organic Se as selenocystathionine. Cappa *et al*. (2015) found 75% organic Se in *S. elata*, which is similar to that in nonhyperaccumulator *S. albescens*, whose organic Se fraction was found to consist of exclusively selenocystathionine (Freeman *et al*., 2010); the same may be the case for *S. elata*.

Selenium likely causes oxidative stress (Van Hoewyk, 2013) and has been reported to induce the expression of genes coding for peroxidases and GSH‐related enzymes (Freeman *et al*., 2010; Rios *et al*., 2009). The results from this study indicate that the Se hyperaccumulator has elevated antioxidant scavenging capacity, which may contribute to its Se tolerance. Several genes involved in GSH synthesis (*gsh1*) and conjugation (*gstf7*), and in free radical scavenging via peroxidase activity (*gpx6*,* apx1* and *tpx1*) were expressed much more highly in *S. pinnata* than *S. elata*. In fact, the peroxidase family category (in Mapman) had the highest proportion of genes with greater expression in *S. pinnata* than *S. elata* for almost all treatments analysed. This explains the higher GSH levels found in *S. pinnata* compared to the nonhyperaccumulator *S. albescens* (Freeman *et al*., 2010), and the fact that when supplied with Se, there were lower levels of ROS accumulated in *S. pinnata* than *S. albescens*. It is possible that constitutively elevated levels of GSH and peroxidases ‘prime’ *S. pinnata* for oxidative stress. The hyperaccumulators appear to be similarly ‘primed’ for biotic stress, judged from their elevated expression levels of defence‐related genes. Some of these responses may be triggered by shared upstream signalling pathways. Three plant hormones that have been implicated before to play a role in Se tolerance and defence are JA, SA and ethylene. Two macroarray analyses using the same set of ∼350 genes found expression levels of defence‐related genes to be constitutively higher in *S. pinnata* compared to *S. albescens*, and more induced by Se in Se‐resistant than Se‐sensitive *A. thaliana* accessions with ethylene and JA restored Se tolerance (Tamaoki *et al*., 2008a), and application of JA to *S. pinnata* increased leaf Se levels (Freeman *et al*., 2010). Moreover, tissue levels of JA and SA were higher in *S. pinnata* than *S. albescens* (Freeman *et al*., 2010). The elevated expression of JA biosynthesis genes found here (especially *lox2* and *lox6*) can explain the previously identified elevated JA levels in *S. pinnata*. *Lox2* was reported by Tamaoki *et al*. (2008a) to be induced by Se in Se‐resistant *A. thaliana* accessions, and *lox6* is currently the only lipoxygenase thought to positively regulate JA levels in roots as well as shoots (Grebner *et al*., 2013). Elevated JA levels can explain the higher expression levels of GSH biosynthesis genes in the Se tolerant species. In *A. thaliana* Xiang and Oliver (1998) showed that JA was primarily responsible for increasing the transcript levels of *gsh1*,* gsh2* and *gr1*. Jasmonic acid as well as ethylene has been reported to induce S assimilation (Tamaoki *et al*., 2008a) and thus may be responsible for the observed elevated levels of sulphate transporter and S assimilatory genes.

The greater expression of SA biosynthesis genes such as *ics2* and *pal*, as well as the SA‐responsive genes such as transcription factors *wrky* and *tga*,* eps* (enhanced *Pseudomonas* susceptibility) and pathogenesis related (*pr)* in the Se hyperaccumulator indicate that SA is involved in Se hyperaccumulation as well, which agrees with earlier findings. Tamaoki *et al*. (2008a) found increased SA levels in *A. thaliana* following Se treatment. A potential upstream signalling gene for the SA‐mediated responses is mildew resistance locus (*mlo*)*12*, whose transcript was up to 70‐fold more abundant in *S. pinnata* than *S. elata* roots. MLO interacts with calmodulin (Kim *et al*., 2002), the expression of which was found by Tamaoki *et al*. (2008a) to be induced by Se in *A. thaliana*. Incidentally, calmodulin 3 was also 10‐fold more highly expressed in *S. pinnata* than *S. elata* (~2500 vs ~250 RPKM).

Ethylene levels have been reported to induce phenylalanine ammonia‐lyase (PAL) activity (Chalutz, 1973), which in turn may be involved in SA synthesis. The Se hyperaccumulator showed higher expression of ethylene‐responsive gene *ein3* and as well as the gene encoding mitogen‐activated protein kinase 6 (*mapk6)*, a key protein in ethylene signalling. Ethylene and JA may cooperatively induce the expression of *pdf* (Leon‐Reyes *et al*., 2010; Penninckx *et al*., 1998), a defence‐associated gene found to be more expressed in *S. pinnata* compared to *S. albescens* (Freeman *et al*., 2010). In *A. thaliana* (Tamaoki *et al*., 2008b), *pdf* was more induced in tolerant accessions by Se treatment; furthermore, plants that overexpressed *A. halleri pdf 1.1* showed a significant increase in tolerance to Se compared to wild‐type. We also found higher expression levels of *pdf 1.1* and *pdf 1.2C* in the Se hyperaccumulator. It is possible that *pdf* contributes to Se tolerance via an unknown mechanism.

Overall, the results from this comparative Se‐dependent transcriptome analysis agreed well with earlier findings obtained from biochemical and macroarray studies and also provide new insights into the Se hyperaccumulation and tolerance mechanisms in *S. pinnata*. We propose several potential Se‐sensing and signalling genes that may trigger the induction of these defence hormone biosynthesis genes, and merit further study. Ideally, a ‘key gene’ maybe identified that triggers the cascade of events that leads to the Se hyperaccumulation syndrome. Such a gene would be very promising for the genetic engineering of plants with superior capacity for Se accumulation and tolerance, which would have wide applications in phytoremediation and biofortification.

Several interesting parallels emerge from this study, between the hyperaccumulation mechanisms in this Se hyperaccumulator and those in metal hyperaccumulating Brassicaceae. In *Noccaea caerulescens*, Fones *et al*. (2013) found defence signalling through ROS to be up‐regulated by Zn, but not anymore by pathogen infection. They suggested that, as metal hyperaccumulation started functioning as elemental defence, normal defence responses became progressively uncoupled from ROS signalling. In other recent studies on *A. halleri*, Stolpe *et al*. (2017a,b) found that Zn and Cd levels were co‐up‐regulated with the organic defence compounds glucosinolates upon herbivore attack, indicating that the metals have not replaced these alternative defence compounds in this hyperaccumulator. Further studies are needed for *S. pinnata* to investigate whether its apparent up‐regulation of defence signalling translates to some degree of enhanced pathogen/herbivore protection also in the absence of Se, and whether/how Se accumulation is coordinated with levels of glucosinolates.

## Experimental procedures

### Plant growth

Seeds of *S. pinnata* (Western Native Seed, Coaldale, CO) and *S. elata* (collected in NV, 38°11′36″N 117°59″15″W) were surface‐sterilized and stratified for 48 h at 4 °C. Seeds were germinated on sterile petri dishes and transferred to sealed Phytatrays^TM^ (Sigma‐Aldrich, St. Louis, MO) on solid "strength MS basal salts medium (Murashige and Skoog, 1962) with 1% sucrose and 0 or 20 μm sodium selenate. It is relevant to mention that half‐strength MS medium contains 750 μm sulphate, and no Se. Plants were incubated in a growth chamber at a light intensity of 150 μmol photons per m^2^ per s with a 16/8 L/D photoperiod at 23 °C. Three plants were grown per container, with three containers per species and treatment. After 30 days, one plant per container was harvested and the roots rinsed to remove external Se. The plants were separated into root and shoot and frozen in liquid nitrogen for RNA sequencing (three bioreplicates per treatment). The remaining two plants from each container were harvested, rinsed, separated into roots and shoots and weighed.

### RNA sequencing

Frozen plant samples (24 in total: two species, two organs, two Se treatments, *n* = 3) were shipped to the University of Missouri where total RNA was extracted using an RNA Mini Kit (Invitrogen, Carlsbad, CA). The mRNA was purified and used to construct Illumina cDNA libraries using the TruSeq RNA Kit, then sequenced on an Illumina HiSeq‐2000 at the University of Missouri's DNA Core Facility. Paired‐end 100 bp sequencing was performed for one biological replicate for each species–organ–Se treatment combination (eight samples). The sequences were quality filtered using NextGENe ver. 2.3.1 (SoftGenetics, State College, PA), removing adapter sequences, reads with a median quality score of <22, trimmed reads at positions that had three consecutive bases with a quality score of <20, and any trimmed reads with a total length <40 bp. This resulted in 77.7% of the over 500.7 million sequenced reads passing the quality‐score filter. The quality‐filtered data were used to assemble *de novo* transcript contiguous sequences (contigs) for each species separately using Trinity ver. 2011‐11‐26 (Grabherr *et al*., 2011) with default parameters, which is considered to be superior to other methods (Honaas *et al*., 2016).

Contigs shorter than 300 bp were removed. Next, single‐end 50 bp sequencing was performed on the remaining two biological replicates for each species–organ–Se treatment combination (16 additional samples) generating an additional 627.7 million reads. Quality‐filtered reads for all three biological replicates were aligned to the *de novo* assemblies using NextGENe ver. 2.3.1 and counting only uniquely mapped reads, using parameters (A. Matching Requirement: ≥40 Bases and ≥98%, B. Allow Ambiguous Mapping: FALSE, and C. Rigorous Alignment: TRUE) resulting in the alignment of ~43.7% of all filtered reads.

Contigs were annotated using BLASTn against the *Arabidopsis thaliana* cDNA database (TAIR) and assigned homologs with an e‐value threshold of 0.00005. *Arabidopsis thaliana* was used as a reference because it has a fully annotated genome, and is in the same family (Brassicaceae) as *Stanleya*. The genetic divergence between the two *Stanleya* species is expected to be sufficiently small to not pose a problem. According to Edger *et al*. (2015) and Hohmann *et al*. (2015) the divergence between the two *Stanleya* species is likely to be 2–3 MYA, whereas the divergence between *Arabidopsis* and *Stanleya* is around 23 MYA. Indeed, average nucleotide identity between *A. thaliana* and each of the *Stanleya* species (based on three full length cDNAs) was the same at 87.5%; between the *Stanleya* species, it was 96% (Pilon‐Smits, unpublished results).

Contigs annotated to the same ATID (*A. thaliana* identification) were associated with one gene and their RPKM (reads per kilobase per million mapped reads) values were summed. As a standard preliminary step, we employed the nonspecific filtering procedure to remove those genes with low overall intensity or variability. Specifically, we employed both the intensity‐based and variability‐based criteria as follows. A gene *r* with negligible expression levels across all 24 samples was excluded if $r\in S_{1}=\{g:max_{\mathrm{ijl}}\sum_{k=1}^{3}Z_{g,ijkl}/3<\varepsilon_{1}\}$ where *Z* stands for RPKM values and *ɛ*_1_ is chosen to be the 2% grand quantiles of sample means. Subscript letters refer to gene (*g *=* *1, 2, … 19 129), organ (*l *=* *1, 2 for root and shoot, respectively), biological replicate (*k *=* *1, 2, 3), plant species (*i *=* *1, 2 for *S. elata* and *S. pinnata*, respectively) and treatment (*j *=* *1, 2 for 0 and 20 μm Se, respectively).

In addition, genes that essentially did not have any within‐sample variations were excluded, that is gene r was removed if $r\in S_{2}=\left\{g:max_{\mathrm{ijl}}\sum_{k=1}^{3}{\left(Z_{g,ijkl}-\sum_{k=1}^{3}Z_{g,ijkl}/3\right)}^{2}<\varepsilon_{2}\right\}$ where *ɛ*_2_ was selected to be the 2% grand quantiles of sample variations, as before. The retained genes were further analysed for differential expression. Additional text describing statistical analysis can be found in Appendix S1.

### Mapman visualization

The estimated effects based on model parameters for significant differentially expressed (*q* < 0.005) genes were further visualized using Mapman ver. 3.5.1 (Thimm *et al*., 2004; Usadel *et al*., 2005). A total of 12 analysis files were generated (https://www.dropbox.com/sh/5fo1ikp4ca7my4x/AAD0S_lVuggP6I7WkXkx4WA0a?dl=0). Within Mapman, the mapping tool ATh_AGI_ISOFORM_MODEL_TAIR10_Aug2012 was used to visualize genes in pathways. Bins were identified that were significantly different (*P* < 0.05) from others within the same pathway, as well as significantly differentially expressed genes between *S. pinnata* and *S. elata* within each bin (Usadel *et al*., 2005). For genes that were differentially expressed between species for all combinations of organ and Se treatment in selected bins, we calculated which proportion of genes were more expressed in one or the other plant species.

### Gene expression via quantitative real‐time PCR

Root RNA was extracted from *S. pinnata* and *S.elata* plants (three biological replicates) grown in hydroponics for 2 weeks in "strength Hoagland solution containing 0.5 mm S and then treated for 3 days with 20 μm Se or without. Experiments were performed as described in Schiavon *et al*. (2015). Primer pairs used are listed in Table S3. The CT values were analysed using the Q‐gene software (Muller *et al*., 2002).

### Elemental analysis

A follow‐up experiment was carried out to measure elemental concentrations and dry weights of plants grown under the same conditions as the samples used for RNA‐seq. Three biological replicates from each treatment were used, each consisting of five plants (*n* = 3). The roots did not yield sufficient biomass for elemental analysis, so they were not analysed. The dried shoot material was acid digested and analysed for Se and S levels using ICP‐OES as described by Cappa *et al*. (2014).

## Conflict of interest

The authors declare no financial or commercial conflict of interest.

## Supporting information


**Appendix S1** Description of the statistical analyses of the RNA sequencing data, comparing effects of treatments, species and organs.
**Figure S1** Biomass production and Se and S accumulation by *Stanleya pinnata* and *S. elata*. The plants were grown from seed on agar with 0 or 20 μm sodium selenate.
**Figure S2** Overview of differential expression between the plant species by organ and Se treatment.
**Figure S3** Reverse‐transcription polymerase chain reaction (RT‐PCR) analysis of root transcript levels.
**Table S1** Top 100 significant (*q*‐value < 0.005) differentially expressed genes in response to Se treatment.
**Table S2** Top 100 significant (*q*‐value < 0.005) differentially expressed genes between species.
**Table S3** List of primers used in qRT‐PCR reactions.
