# Supplementary material for: Transcriptome‐wide comparison of selenium hyperaccumulator and nonaccumulator Stanleya species provides new insight into key processes mediating the hyperaccumulation syndrome
Source: Plant Biotechnol J. 2018 Mar 14;16(9):1582–94. doi: 10.1111/pbi.12897 (PMC6097121; doi:10.1111/pbi.12897)
Supplement: Supplementary file 1 — Appendix S1 Description of the statistical analyses of the RNA sequencing data, comparing effects of treatments, species and organs. Figure S1 Biomass production and Se and S accumulation by Stanleya pinnata and S. elata. The plants were grown from seed on agar with 0 or 20 μm sodium selenate. Figure S2 Overview of differential expression between the plant species by organ and Se treatment. Figure S3 Reverse‐transcription polymerase chain reaction (RT‐PCR) analysis of root transcript levels. Table S1 Top 100 significant (q‐value < 0.005) differentially expressed genes in response to Se treatment. Table S2 Top 100 significant (q‐value < 0.005) differentially expressed genes between species. Table S3 List of primers used in qRT‐PCR reactions. [file PBI-16-1582-s001.docx]

## Supporting Information

**Transcriptome-wide comparison of selenium hyperaccumulator and non-accumulator Stanleya species provides new insight into key processes mediating the hyperaccumulation syndrome**

Authors: Jiameng Wang, Jennifer J. Cappa, Jonathan Harris, Patrick P. Edger, Wen Zhou, J. Chris Pires, Michael Adair, Sarah Unruh, Mark P. Simmons, Michela Schiavon, Elizabeth A.H. Pilon-Smits

The following Supporting Information is available for this article:

**Supplemental text 1 (to Methods section)** Description of the statistical analyses of the RNA sequencing data, comparing effects of treatments, species and organs.

As an initial statistical analysis, edgeR and DESeq generalized linear models were used to identify differentially expressed gene contigs between Se treatments within species and organs (results not shown). For more extensive statistical analysis, the RPKM values were normalized using the trimmed mean of M-values (TMM) procedure [1,2]. The normalized RPKMs were further transformed by variance stabilization transformations [3]. The experiment naturally follows a split-plot design, and to account the correlations among samples within the same combination of species and treatment, we employed the linear mixed model for analysis of the processed libraries [4]. The model is described as following:

$$Y_{g,ijkl}=\mu_{g}+\alpha_{g,i}+\beta_{g,j}+\left( \alpha\beta\right)_{g,ij}+e_{g,ijk}+\gamma_{g,l}+\left( \alpha\gamma\right)_{g,il}+\left( \beta\gamma\right)_{g,jl}+\left( \alpha\beta\gamma\right)_{g,ijl}+\varepsilon_{g,ijkl}$$

where *Y_g,ijkl_* denotes the processed expression level of the *g^th^* gene (*g* = 1, 2, … 19,129) of the *l^th^* organ (*l* = 1, 2 for root and shoot, respectively) of the *k*^th^ biological replicate (*k* = 1, 2, 3) from the *i^th^* species (*i* = 1, 2 for *S. elata* and *S. pinnata*, respectively) for the *j^th^* treatment (*j* = 1, 2 for 0 and 20 µM Se, respectively). With the sub-index *g* suppressed for simplicity, we summarize the model parameters as follows: *α_i_* models the effect of species; *β_j_* models the treatment effects; (*αβ*)*_ij_* models the interactions between species and treatment; *γ_l_* models the organ effects; (*αγ*)*_il_* models the interactions between species and organ; (*βγ*)*_jl_* models the interactions between treatment and organ; (*αβγ*)*_ijl_* models the 3-way interaction between species, treatment, and organ; $e_{ijk}\sim i. i.d. N\left( 0, \sigma_{e}^{2} \right)$ models correlations among samples within the same combination of species and treatment; and $\varepsilon_{ijkl}\sim i. i.d. N\left( 0, \sigma^{2} \right)$models measurement errors.

Data processing, model fitting and subsequent analyses were conducted using SAS ver. 9.4, R ver. 3.1.2, and Bioconductor ver. 3.1 [5]. The above-described linear mixed model was fitted using the SAS procedure *proc mixed* to obtain estimated model parameters and test hypotheses of biological interest. For example, using the *proc mixed* procedure, we estimated for each gene the main effects of the (Se) treatment and species as well as the simple effects of treatment for each species, and obtained the corresponding p-values for testing hypotheses regarding the significance of these effects, such as whether or not the main effect of treatment or species is significant. Similarly, the effect of treatment, segregated by organ type for each species; the effect of species for each organ type, segregated by treatments; and the interaction effect of species on the differences between treatments were estimated and tested for each gene for a more detailed analysis. Corresponding p-values for testing the significance of these estimated effects are also obtained for each gene using the *proc mixed* procedure and associated q-values were derived. The Benjamini-Hochberg procedure was employed for controlling the false discovery rate (FDR) at the 0.005 level [6]. Then, for each of the above hypotheses of biological interest, genes were declared significant by the above general linear hypothesis testing procedure with FDR controlled at the 0.005 level. For example, the gene whose ATID is AT1G01030.1 has a q-value for the effect of Se treatment < 0.005 and is therefore considered a differentially expressed (DE) gene for the effect of Se treatment, whose 0 and 20 µM Se treatment effects were estimated by corresponding model parameters$.$

*References*

[1] Oshlack; A.; Robinson; M.D.; Young M.D.; From RNA-seq reads to differential expression results. Genome Biol. 2010; 11; 200.

[2] Robinson; M.D.; Oshlack; A.; A scaling normalization method for differential expression analysis of RNA-seq data. Genome Biol. 2010; 11:R25.

[3] Durbin; B.P.; Hardin; J.S.; Hawkins; D.M.; Rocke; D.N.; A variance-stabilizing transformation for gene-expression microarray data. Bioinformatics. 2002; 18; 105-110.

[4] Morris; M.D.; Design of Experiments: An Introduction Based on Linear Models. Chapman and Hall/CRC. 2010.

[5] Gentleman; R.C.; Carey; V.J.; Bates; D.M.; Bolstad; B.; et al.; Bioconductor: open software development for computational biology and bioinformatics. Genome Biol.; 2004; 5:R80.

[6] Benjamini; Y; Hochberg; Y.; Controlling the false discovery rate; a practical and powerful approach to multiple testing. J. Royal Stat. Soc. (Series B). 1995; 57; 289-300.

**A.**

**B.**

**C.**

**D.** **
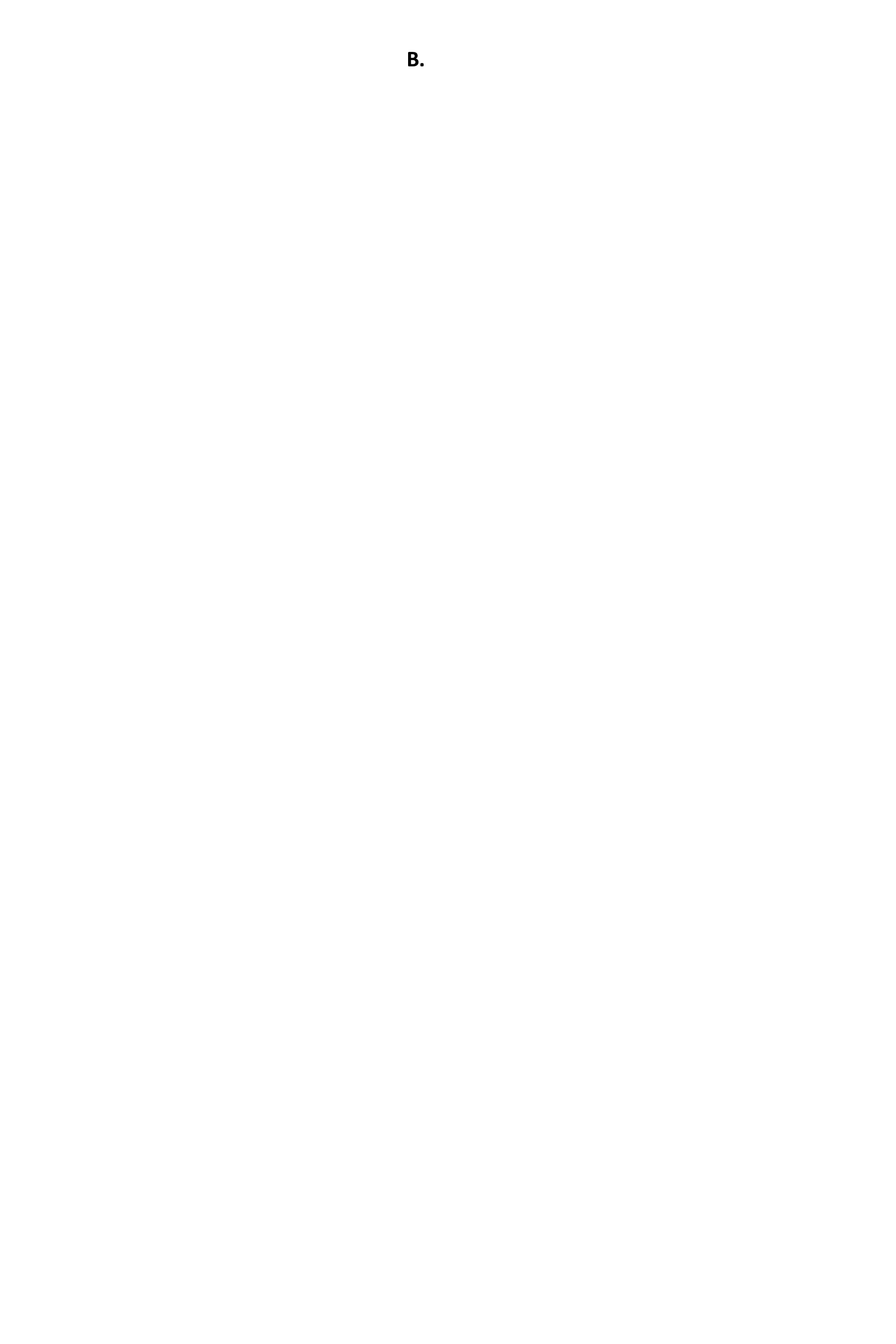
**


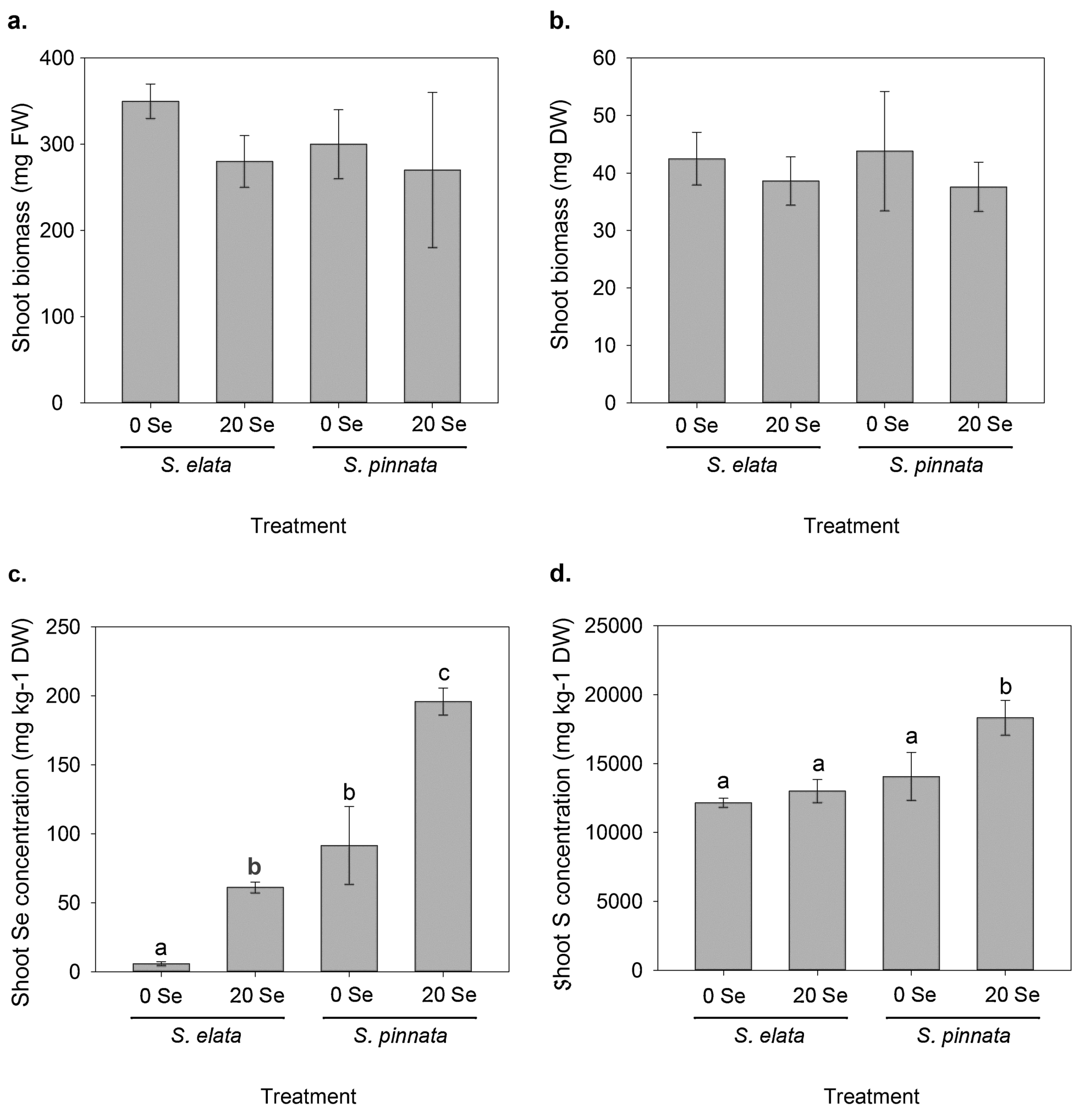


**Figure S1** Biomass production and Se and S accumulation by *S. pinnata* and *S. elata*. The plants were grown from seed on agar with 0 or 20 µM sodium selenate. **(A)** Shoot fresh weight of plants used for transcriptome analysis. **(B)** Shoot dry weight of plants grown for elemental analysis. **(C)** Shoot Se concentration. **(D)** Shoot S concentration. Values shown represent the mean of three replicates + SEM. Letters above bars indicate significant differences (p<0.05) between treatments using ANOVA with Tukey-Kramer post hoc analysis.

**
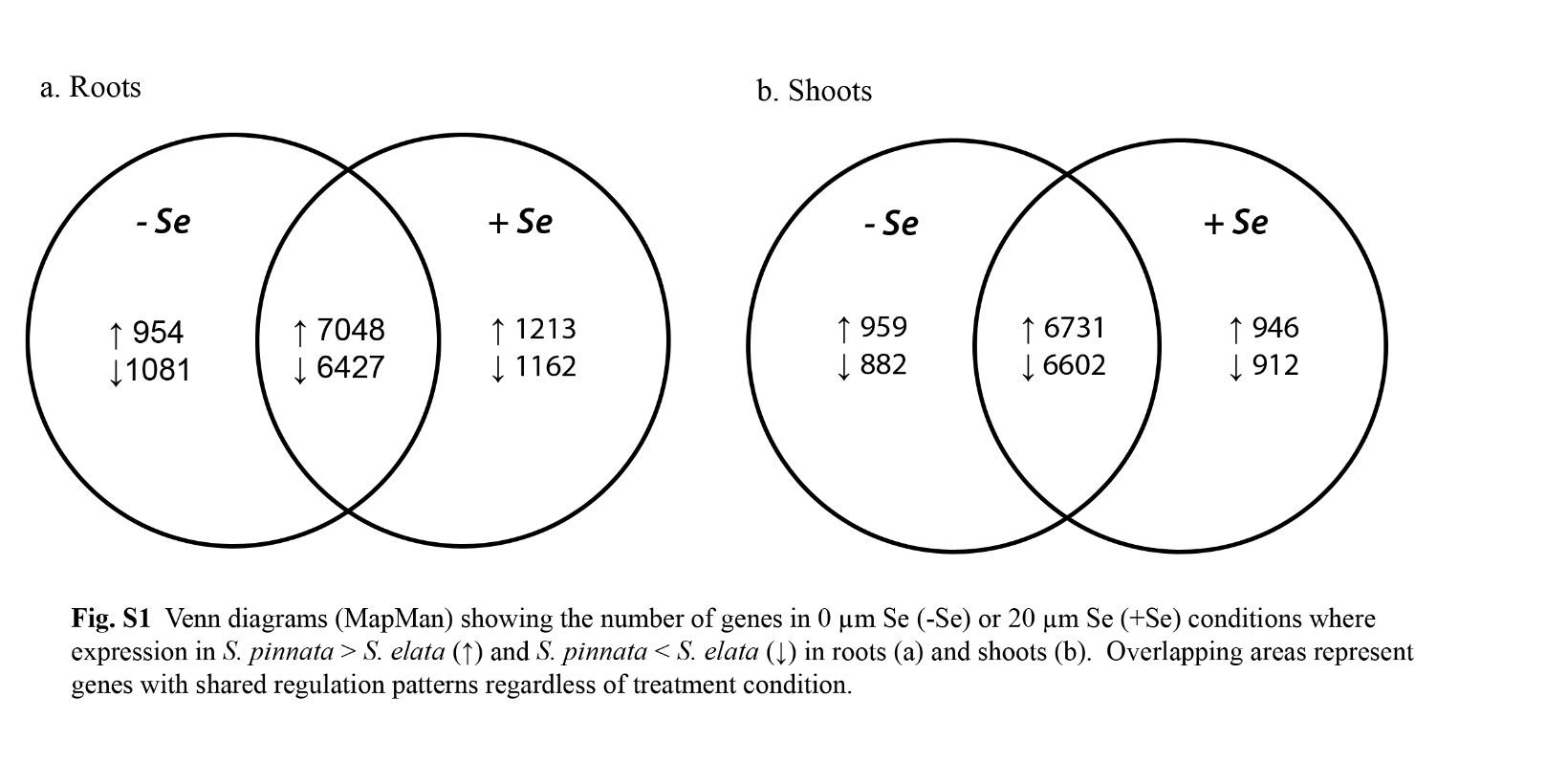
**

**B. Shoots**

**A. Roots.**

**Figure S2** Overview of differential expression between the plant species by organ and Se treatment. Venn diagrams show the number of genes in 0 µm Se (-Se) or 20 µm Se (+Se) conditions where expression in *S. pinnata* > *S. elata* (↑) and *S. elata* > *S. pinnata* (↓) in roots (**A**) and shoots (**B**). Overlapping areas show genes with shared regulation patterns regardless of treatment condition

**Figure S3.** Reverse-transcription polymerase chain reaction (RT-PCR) analysis of root transcript levels in *Stanleya pinnata* and *Stanleya elata* grown in hydroponics on half-strength Hoagland’s solution (0.5 mM S, no Se). The plants were analyzed as described in Schiavon et al. (2015), using the described primer sets for *Sultr1;2, APS1*, *APS2* and actin. Quantitative RT-PCR analyses were performed on three biological replicates, with two technical replicates. All quantifications were normalized to the actin housekeeping gene and amplified in the same conditions. The obtained CT values were analyzed with the Q-gene software by averaging three independently calculated normalized expression values for each sample. Expression values are given as the mean of the normalized expression values of the biological triplicates, calculated according to Eq. 2 of the Q-gene software (Muller et al., 2002).

**Table S1.** Top 100 significant (q-value < 0.005) differentially expressed genes in response to Se treatment. **(a)** *S. elata* roots, **(b)** *S. pinnata* roots, **(c)** *S. elata* shoots, **(d)** *S. pinnata* shoots. Fold difference is calculated as the RPKM ratio of 0 µM/20 µM Se. Effect refers to effect of magnitude. (+) or (-) values indicate increased or decreased gene expression with Se treatment, respectively. The larger the absolute value of the effect is, the greater the treatment effect. Effect values were used to separate genes based on expression direction in descending order, with the most differentially expressed on top.

a. *S. elata* roots

| **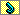ATID** | **fold difference** | **effect** | **annotation** |
| --- | --- | --- | --- |
| **Increased expression** | | | |
| **AT2G07709.1** | 13.08 | 2.24 | pseudogene, similar to NADH dehydrogenase, |
| **AT2G07717.1** | 18.32 | 2.04 | pseudogene, similar to NADH-ubiquinone |
| **AT2G07711.1** | 20.23 | 1.69 | pseudogene, similar to NADH dehydrogenase subunit 5 |
| **AT2G07727.1** | 18.96 | 1.53 | Di-haem cytochrome, transmembrane; Cytochrome b/b6, C-terminal |
| **AT2G07733.1** | 18.44 | 1.51 | pseudogene, similar to NADH dehydrogenase subunit 2 |
| **AT2G47230.2** | 21.67 | 1.15 | DOMAIN OF UNKNOWN FUNCTION 724 6 (DUF6) |
| **AT1G10890.1** | 3.15 | 1.13 | unknown protein |
| **AT2G19110.1** | 2.48 | 1.00 | heavy metal atpase 4 (HMA4) |
| **AT5G40170.1** | 3.92 | 0.98 | receptor like protein 54 (RLP54) |
| **AT2G36420.1** | 2.23 | 0.94 | unknown protein |
| **AT5G49930.1** | 2.42 | 0.90 | embryo defective 1441 (emb1441) |
| **AT3G54010.2** | 4.42 | 0.90 | PASTICCINO 1 (PAS1) |
| **AT1G65860.1** | 2.06 | 0.89 | flavin-monooxygenase glucosinolate S-oxygenase 1 (FMO GS-OX1) |
| **AT3G14460.1** | 3.83 | 0.89 | LRR and NB-ARC domains-containing disease resistance protein |
| **AT2G07734.1** | 18.89 | 0.88 | Alpha-L RNA-binding motif/Ribosomal protein S4 family protein |
| **AT2G07783.1** | 15.61 | 0.86 | pseudogene, similar to Ccl1 |
| **AT2G17430.1** | 33.59 | 0.85 | MILDEW RESISTANCE LOCUS O 7 (MLO7) |
| **AT2G07712.1** | 22.90 | 0.82 | pseudogene, similar to maturase-related protein |
| **AT5G25310.1** | 6.98 | 0.82 | Exostosin family protein |
| **AT1G10320.1** | 2.54 | 0.82 | Zinc finger C-x8-C-x5-C-x3-H type family protein |
| **AT4G07668.1** | 2.80 | 0.81 | gypsy-like retrotransposon family |
| **AT1G16440.1** | 33.05 | 0.78 | root hair specific 3 (RSH3) |
| **AT3G19670.1** | 1.94 | 0.77 | pre-mRNA-processing protein 40B (PRP40b) |
| **AT3G57120.1** | 10.60 | 0.76 | Protein kinase superfamily protein |
| **AT5G23110.1** | 2.01 | 0.76 | Zinc finger, C3HC4 type (RING finger) family protein |
| **AT3G19190.1** | 2.19 | 0.75 | AUTOPHAGY 2 (ATG2) |
| **AT3G01770.1** | 2.34 | 0.75 | bromodomain and extraterminal domain protein 10 (BET10) |
| **AT5G38383.1** | 8.52 | 0.75 | gypsy-like retrotransposon family (Athila) |
| **AT1G19485.2** | 3.21 | 0.74 | Transducin/WD40 repeat-like superfamily protein |
| **AT1G79950.1** | 2.70 | 0.73 | RAD3-like DNA-binding helicase protein |
| **AT1G06490.1** | 2.78 | 0.73 | glucan synthase-like 7 (GSL07) |
| **AT2G07715.1** | 26.63 | 0.73 | Nucleic acid-binding, OB-fold-like protein |
| **AT3G13790.2** | 2.22 | 0.72 | ATBFRUCT1 |
| **AT1G16460.4** | 3.88 | 0.72 | rhodanese homologue 2 (RDH2) |
| **AT1G54490.1** | 2.99 | 0.72 | exoribonuclease 4 (XRN4) |
| **AT2G07687.1** | 15.51 | 0.72 | Cytochrome c oxidase, subunit III |
| **AT4G02660.1** | 2.18 | 0.72 | Beige/BEACH domain ;WD domain, G-beta repeat protein |
| **AT1G48090.1** | 1.99 | 0.72 | calcium-dependent lipid-binding family protein |
| **AT3G02070.1** | 2.26 | 0.71 | Cysteine proteinases superfamily protein |
| **AT3G20475.1** | 2.69 | 0.70 | MUTS-homologue 5 (MSH5) |
| **Decreased expression** | | | |
| **AT2G22240.2** | 0.16 | -1.28 | myo-inositol-1-phosphate synthase 2 (MIPS2) |
| **AT4G23680.1** | 0.33 | -1.28 | Polyketide cyclase/dehydrase and lipid transport superfamily protein |
| **AT1G50010.1** | 0.35 | -1.25 | tubulin alpha-2 chain (TUA2) |
| **AT5G05170.1** | 0.36 | -1.23 | CONSTITUTIVE EXPRESSION OF VSP 1 (CEV1) |
| **AT5G25980.3** | 0.33 | -1.18 | glucoside glucohydrolase 2 (TGG2) |
| **AT1G52000.1** | 0.39 | -1.17 | Mannose-binding lectin superfamily protein |
| **AT3G20370.1** | 0.35 | -1.15 | TRAF-like family protein |
| **AT4G11320.1** | 0.36 | -1.10 | Papain family cysteine protease |
| **AT2G33070.2** | 0.27 | -1.09 | nitrile specifier protein 2 (NSP2) |
| **AT5G23020.1** | 0.42 | -1.08 | 2-isopropylmalate synthase 2 (IMS2) |
| **AT3G63200.1** | 0.36 | -1.07 | PATATIN-like protein 9 (PLP9) |
| **AT1G56070.1** | 0.44 | -1.06 | LOW EXPRESSION OF OSMOTICALLY RESPONSIVE GENES 1 (LOS1) |
| **AT3G09260.1** | 0.42 | -1.06 | PYK10 |
| **AT5G56030.2** | 0.35 | -1.05 | heat shock protein 81-2 (HSP81-2) |
| **AT2G30860.2** | 0.31 | -1.05 | glutathione S-transferase PHI 9 (GSTF9) |
| **AT2G37040.1** | 0.37 | -1.03 | PHE ammonia lyase 1 (PAL1) |
| **AT4G32410.1** | 0.46 | -1.01 | cellulose synthase 1 (CESA1) |
| **AT2G38080.1** | 0.19 | -0.99 | IRREGULAR XYLEM 12 (IRX12) |
| **AT1G48760.2** | 0.38 | -0.98 | delta-adaptin (delta-ADR) |
| **AT5G12250.1** | 0.41 | -0.98 | beta-6 tubulin (TUB6) |
| **AT4G05050.1** | 0.43 | -0.97 | ubiquitin 11 (UBQ11) |
| **AT1G02500.2** | 0.41 | -0.96 | S-adenosylmethionine synthetase 1 (SAM1) |
| **AT1G15690.2** | 0.35 | -0.91 | AVP1 |
| **AT1G28400.1** | 0.47 | -0.90 | unknown protein |
| **AT3G53260.1** | 0.38 | -0.89 | phenylalanine ammonia-lyase 2 (PAL2) |
| **AT4G13940.3** | 0.47 | -0.87 | HOMOLOGY-DEPENDENT GENE SILENCING 1 (HOG1) |
| **AT2G01520.1** | 0.54 | -0.86 | MLP-like protein 328 (MLP328) |
| **AT3G60750.2** | 0.43 | -0.86 | Transketolase |
| **AT3G03040.1** | 0.11 | -0.85 | F-box/RNI-like superfamily protein |
| **AT1G52400.3** | 0.32 | -0.84 | beta glucosidase 18 (BGLU18) |
| **AT1G54040.2** | 0.46 | -0.84 | epithiospecifier protein (ESP) |
| **AT5G44340.1** | 0.41 | -0.82 | tubulin beta chain 4 (TUB4) |
| **AT3G16470.3** | 0.55 | -0.81 | JASMONATE RESPONSIVE 1 (JR1) |
| **AT1G51680.3** | 0.38 | -0.81 | 4-coumarate:CoA ligase 1 (4CL1) |
| **AT1G78120.1** | 0.46 | -0.81 | Tetratricopeptide repeat (TPR)-like superfamily protein |
| **AT4G38770.1** | 0.46 | -0.80 | proline-rich protein 4 (PRP4) |
| **AT5G67360.1** | 0.53 | -0.80 | ARA12; FUNCTIONS IN: serine-type endopeptidase activity |
| **AT3G47470.1** | 0.52 | -0.80 | light-harvesting chlorophyll-protein complex I subunit A4 (LHCA4) |
| **AT4G13930.1** | 0.41 | -0.80 | serine hydroxymethyltransferase 4 (SHM4) |
| **AT1G07890.6** | 0.39 | -0.79 | ascorbate peroxidase 1 (APX1) |
| **AT4G18780.1** | 0.29 | -0.79 | IRREGULAR XYLEM 1 (IRX1) |
| **AT1G48110.2** | 0.48 | -0.78 | evolutionarily conserved C-terminal region 7 (ECT7) |
| **AT1G44575.2** | 0.49 | -0.78 | NONPHOTOCHEMICAL QUENCHING 4 (NPQ4) |
| **AT5G38480.2** | 0.46 | -0.77 | general regulatory factor 3 (GRF3_ |
| **AT5G17920.2** | 0.53 | -0.77 | COBALAMIN-INDEPENDENT METHIONINE SYNTHASE (ATCIMS) |
| **AT5G44790.1** | 0.47 | -0.77 | RESPONSIVE-TO-ANTAGONIST 1 (RAN1) |
| **AT3G15950.1** | 0.24 | -0.75 | NAI2 |
| **AT5G07030.1** | 0.49 | -0.75 | Eukaryotic aspartyl protease family protein |
| **AT2G38120.1** | 0.51 | -0.75 | AUXIN RESISTANT 1 (AUX1) |
| **AT2G41840.1** | 0.53 | -0.74 | Ribosomal protein S5 family protein |
| **AT4G21960.1** | 0.57 | -0.74 | PRXR1 |
| **AT3G16240.1** | 0.32 | -0.74 | delta tonoplast integral protein (DELTA-TIP) |
| **AT3G16410.1** | 0.20 | -0.74 | nitrile specifier protein 4 (NSP4) |
| **AT4G33090.1** | 0.45 | -0.74 | aminopeptidase M1 (APM1) |
| **AT4G03050.1** | 0.42 | -0.73 | AOP3 |
| **AT1G73330.1** | 0.19 | -0.71 | drought-repressed 4 (DR4) |
| **AT5G17420.1** | 0.23 | -0.71 | IRREGULAR XYLEM 3 (IRX3) |
| **AT3G03780.3** | 0.60 | -0.71 | methionine synthase 2 (MS2) |
| **AT2G36880.2** | 0.44 | -0.71 | methionine adenosyltransferase 3 (MAT3) |

b. *S. elata* shoots

| ATID | fold difference | effect | annotation |
| --- | --- | --- | --- |
| Increased expression | | | |
| AT1G16460.4 | 3.63 | 0.79 | rhodanese homologue 2 (RDH2) |
| AT2G47230.2 | 9.19 | 0.75 | DOMAIN OF UNKNOWN FUNCTION 724 6 (DUF6) |
| AT2G17430.1 | 93.25 | 0.69 | MILDEW RESISTANCE LOCUS O 7 (MLO7) |
| AT2G23000.1 | 1.94 | 0.65 | serine carboxypeptidase-like 10 (scpl10) |
| AT4G07668.1 | 2.54 | 0.60 | gypsy-like retrotransposon family |
| AT4G37330.1 | 2.97 | 0.59 | cytochrome P450, family 81, subfamily D, polypeptide 4 (CYP81D4) |
| AT5G01595.1 | 3.69 | 0.59 | Potential natural antisense gene |
| AT2G38230.1 | 35.45 | 0.53 | pyridoxine biosynthesis 1.1 (PDX1.1) |
| AT4G24120.1 | 1.81 | 0.51 | YELLOW STRIPE like 1 (YSL1) |
| AT4G25100.5 | 1.63 | 0.47 | Fe superoxide dismutase 1 (FSD1) |
| AT5G56870.1 | 1.48 | 0.47 | beta-galactosidase 4 (BGAL4) |
| AT3G23790.1 | 2.57 | 0.46 | acyl activating enzyme 16 (AAE16) |
| AT3G57120.1 | 4.85 | 0.46 | Protein kinase superfamily protein |
| AT1G69990.1 | 2.19 | 0.45 | Leucine-rich repeat protein kinase family protein |
| AT5G43530.1 | 50.85 | 0.44 | Helicase protein with RING/U-box domain |
| AT1G16440.1 | 20.55 | 0.44 | root hair specific 3 (RSH3) |
| AT5G48320.1 | 12.54 | 0.43 | Cysteine/Histidine-rich C1 domain family protein |
| AT1G27110.3 | 2.03 | 0.43 | Tetratricopeptide repeat (TPR)-like superfamily protein |
| AT5G13630.2 | 1.36 | 0.42 | GENOMES UNCOUPLED 5 (GUN5) |
| AT1G29720.1 | 2.05 | 0.42 | Leucine-rich repeat transmembrane protein kinase |
| AT5G04460.2 | 1.93 | 0.42 | RING/U-box superfamily protein |
| AT4G14370.1 | 59.09 | 0.41 | Disease resistance protein (TIR-NBS-LRR class) family |
| AT1G07110.1 | 1.55 | 0.41 | fructose-2,6-bisphosphatase (F2KP) |
| AT3G56090.1 | 3.12 | 0.41 | ferritin 3 (FER3) |
| AT5G61820.1 | 1.63 | 0.41 | FUNCTIONS IN: molecular_function unknown |
| AT2G31660.1 | 1.56 | 0.39 | SUPER SENSITIVE TO ABA AND DROUGHT2 (SAD2) |
| AT5G43060.1 | 1.43 | 0.39 | Granulin repeat cysteine protease family protein |
| AT5G10180.1 | 2.36 | 0.39 | slufate transporter 2 |
| AT1G79460.1 | 3.07 | 0.39 | GA REQUIRING 2 (GA2) |
| AT1G53510.1 | 2.85 | 0.39 | mitogen-activated protein kinase 18 (MPK18) |
| AT5G02100.1 | 3E+11 | 0.39 | UNFERTILIZED EMBRYO SAC 18 (UNE18) |
| AT3G19170.2 | 1.40 | 0.39 | presequence protease 1 (PREP1) |
| AT1G36160.2 | 1.47 | 0.39 | acetyl-CoA carboxylase 1 (ACC1) |
| AT1G58290.1 | 2.04 | 0.38 | HEMA1 |
| AT3G26770.1 | 2.73 | 0.38 | NAD(P)-binding Rossmann-fold superfamily protein |
| AT1G02640.1 | 1.52 | 0.38 | beta-xylosidase 2 (BXL2) |
| AT4G03050.1 | 1.42 | 0.37 | AOP3 |
| AT3G11930.4 | 1.53 | 0.37 | Adenine nucleotide alpha hydrolases-like superfamily protein |
| AT5G45650.1 | 1.46 | 0.37 | subtilase family protein |
| Decreased expression | | | |
| AT2G14247.1 | 0.15 | -1.55 | Expressed protein |
| AT3G20370.1 | 0.43 | -0.95 | TRAF-like family protein |
| AT5G26000.1 | 0.52 | -0.79 | thioglucoside glucohydrolase 1 (TGG1) |
| AT3G03040.1 | 0.16 | -0.76 | F-box/RNI-like superfamily protein |
| AT3G16470.3 | 0.51 | -0.75 | JASMONATE RESPONSIVE 1 (JR1) |
| AT1G67870.1 | 0.57 | -0.70 | glycine-rich protein |
| AT5G67370.1 | 0.46 | -0.65 | Protein of unknown function (DUF1230) |
| AT5G59320.1 | 0.40 | -0.63 | lipid transfer protein 3 (LTP3) |
| AT3G23800.1 | 0.56 | -0.62 | selenium-binding protein 3 (SBP3) |
| AT5G53450.2 | 0.36 | -0.61 | OBP3-responsive gene 1 (ORG1) |
| AT1G17990.2 | 0.27 | -0.61 | FMN-linked oxidoreductases superfamily protein |
| AT4G14365.1 | 0.32 | -0.61 | XB3 ortholog 4 in Arabidopsis thaliana (XBAT34) |
| AT2G37180.1 | 0.61 | -0.61 | RESPONSIVE TO DESICCATION 28 (RD28) |
| AT1G31580.1 | 0.11 | -0.60 | ECS1 |
| AT5G63520.1 | 0.50 | -0.60 | CONTAINS InterPro DOMAIN/s: F-box domain, Skp2-like |
| AT5G05250.1 | 0.40 | -0.59 | unknown protein |
| AT3G63200.1 | 0.47 | -0.57 | PATATIN-like protein 9 (PLP9) |
| AT3G27060.1 | 0.46 | -0.54 | TSO MEANING 'UGLY' IN CHINESE 2 (TSO2) |
| AT3G21180.1 | 0.28 | -0.54 | autoinhibited Ca(2+)-ATPase 9 (ACA9) |
| AT1G48110.2 | 0.52 | -0.53 | evolutionarily conserved C-terminal region 7 (ECT7) |
| AT1G48760.2 | 0.58 | -0.53 | delta-adaptin (delta-ADR) |
| AT1G22690.3 | 0.44 | -0.53 | Gibberellin-regulated family protein |
| AT4G00780.1 | 0.61 | -0.52 | TRAF-like family protein |
| AT5G25980.3 | 0.68 | -0.51 | glucoside glucohydrolase 2 (TGG2) |
| AT4G04610.1 | 0.66 | -0.50 | APS reductase 1 (APR1) |
| AT1G62420.1 | 0.22 | -0.50 | Protein of unknown function (DUF506) |
| AT1G58270.1 | 0.34 | -0.49 | ZW9 |
| AT5G24660.1 | 0.27 | -0.48 | RESPONSE TO LOW SULFUR 2 (LSU2) |
| AT3G17390.1 | 0.62 | -0.48 | METHIONINE OVER-ACCUMULATOR 3 (MTO3) |
| AT1G68890.1 | 0.69 | -0.48 | magnesium ion binding |
| AT3G16240.1 | 0.61 | -0.48 | delta tonoplast integral protein (DELTA-TIP) |
| AT5G19120.1 | 0.60 | -0.47 | Eukaryotic aspartyl protease family protein |
| AT3G56360.1 | 0.50 | -0.47 | unknown protein |
| AT5G44190.1 | 0.52 | -0.47 | GOLDEN2-like 2 (GLK2) |
| AT3G18290.1 | 0.67 | -0.46 | BRUTUS (BTS) |
| AT2G46660.1 | 0.29 | -0.46 | cytochrome P450, family 78, subfamily A, polypeptide 6 (CYP78A6) |
| AT1G12080.2 | 0.51 | -0.46 | Vacuolar calcium-binding protein-related |
| AT3G51070.1 | 0.09 | -0.44 | S-adenosyl-L-methionine-dependent methyltransferases superfamily protein |
| AT4G16000.1 | 0.34 | -0.44 | unknown protein |
| AT5G04150.1 | 0.37 | -0.43 | BHLH101 |
| AT1G61120.1 | 0.17 | -0.43 | terpene synthase 04 (TPS04) |
| AT1G73602.1 | 0.64 | -0.43 | conserved peptide upstream open reading frame 32 (CPuORF32) |
| AT5G64770.1 | 0.62 | -0.43 | root meristem growth factor 9 (RGF9) |
| AT3G23255.2 | 0.31 | -0.43 | unknown protein |
| AT4G09150.2 | 0.63 | -0.42 | T-complex protein 11 |
| AT1G70850.3 | 0.66 | -0.42 | MLP-like protein 34 (MLP34) |
| AT1G28400.1 | 0.69 | -0.41 | unknown protein |
| AT3G22060.1 | 0.43 | -0.41 | Receptor-like protein kinase-related family protein |
| AT2G31880.1 | 0.57 | -0.41 | SUPPRESSOR OF BIR1 1 (SOBIR1) |
| AT1G20693.3 | 0.63 | -0.41 | high mobility group B2 (HMGB2) |
| AT1G09530.2 | 0.60 | -0.40 | phytochrome interacting factor 3 (PIF3) |
| AT4G21990.2 | 0.53 | -0.40 | APS reductase 3 (APR3) |
| AT5G13740.1 | 0.69 | -0.39 | zinc induced facilitator 1 (ZIF1) |
| AT4G35110.4 | 0.59 | -0.39 | Arabidopsis phospholipase-like protein (PEARLI 4) family |
| AT3G17510.1 | 0.59 | -0.39 | CBL-interacting protein kinase 1 (CIPK1) |
| AT5G52300.2 | 0.53 | -0.38 | LOW-TEMPERATURE-INDUCED 65 (LTI65) |
| AT2G36830.1 | 0.68 | -0.38 | gamma tonoplast intrinsic protein (GAMMA-TIP) |
| AT3G16230.3 | 0.57 | -0.38 | Predicted eukaryotic LigT |
| AT1G64510.1 | 0.70 | -0.37 | Translation elongation  factor EF1B/ribosomal protein S6 family protein |
| AT3G63160.1 | 0.68 | -0.37 | FUNCTIONS IN: molecular_function unknown |
| AT2G37170.1 | 0.62 | -0.37 | plasma membrane intrinsic protein 2 (PIP2b) |

c. *S. pinnata* roots

| ATID | fold difference | effect | annotation |
| --- | --- | --- | --- |
| Increased expression | | | |
| AT4G11310.1 | 37.12 | 1.52 | Papain family cysteine protease |
| AT4G32500.1 | 132.20 | 1.31 | K+ transporter 5 (KT5) |
| AT4G21060.2 | 3.08 | 0.97 | Galactosyltransferase family protein |
| AT5G38100.2 | 5.18 | 0.97 | S-adenosyl-L-methionine-dependent methyltransferases superfamily protein |
| AT5G53010.1 | 3.60 | 0.95 | calcium-transporting ATPase, putative |
| AT4G11320.1 | 43.27 | 0.90 | Papain family cysteine protease |
| AT4G12550.1 | 2.27 | 0.89 | Auxin-Induced in Root cultures 1 (AIR1) |
| AT1G17690.1 | 2.79 | 0.88 | FUNCTIONS IN: molecular_function unknown |
| AT5G48570.1 | 3.11 | 0.87 | FKBP-type peptidyl-prolyl cis-trans isomerase family protein |
| AT2G16060.1 | 2.45 | 0.86 | hemoglobin 1 (HB1) |
| AT1G74590.1 | 2.67 | 0.85 | glutathione S-transferase TAU 10 (GSTU10) |
| AT4G35160.1 | 2.90 | 0.85 | O-methyltransferase family protein |
| AT5G12270.1 | 4.22 | 0.84 | 2-oxoglutarate (2OG) and Fe(II)-dependent oxygenase superfamily protein |
| AT3G12500.1 | 2.12 | 0.81 | basic chitinase (HCHIb) |
| AT2G36120.1 | 2.07 | 0.80 | DEFECTIVELY ORGANIZED TRIBUTARIES 1 (DOT1) |
| AT2G30670.1 | 3.05 | 0.74 | NAD(P)-binding Rossmann-fold superfamily protein |
| AT1G17745.2 | 2.07 | 0.74 | D-3-phosphoglycerate dehydrogenase |
| AT1G78340.1 | 2.37 | 0.68 | glutathione S-transferase TAU 22 (GSTU22) |
| AT4G33110.2 | 4.97 | 0.67 | S-adenosyl-L-methionine-dependent methyltransferases superfamily protein |
| AT3G47730.1 | 1.72 | 0.63 | ATP-binding cassette A2 (ABCA2) |
| AT2G26560.1 | 4.53 | 0.62 | phospholipase A 2A (PLA2A) |
| AT5G43360.1 | 5.77 | 0.61 | phosphate transporter 1 |
| AT4G32950.1 | 4.06 | 0.61 | Protein phosphatase 2C family protein |
| AT1G02920.1 | 1.91 | 0.61 | glutathione S-transferase 7 (GSTF7) |
| AT1G21310.1 | 1.56 | 0.60 | extensin 3 (EXT3) |
| AT5G08260.1 | 3.13 | 0.60 | serine carboxypeptidase-like 35 (scpl35) |
| AT5G22690.1 | 1.75 | 0.59 | Disease resistance protein (TIR-NBS-LRR class) family |
| AT1G52050.1 | 2.64 | 0.59 | Mannose-binding lectin superfamily protein |
| AT1G56430.1 | 2.30 | 0.57 | nicotianamine synthase 4 (NAS4) |
| AT2G43840.1 | 3.05 | 0.57 | UDP-glycosyltransferase 74 F1 (UGT74F1) |
| AT5G36930.2 | 1.81 | 0.57 | Disease resistance protein (TIR-NBS-LRR class) family |
| AT4G19810.1 | 1.67 | 0.57 | Glycosyl hydrolase family protein with chitinase insertion domain |
| AT1G70830.4 | 2.59 | 0.57 | MLP-like protein 28 (MLP28) |
| AT3G47780.1 | 1.80 | 0.56 | ABC2 homolog 6 (ATH6) |
| AT3G61390.2 | 2.38 | 0.56 | RING/U-box superfamily protein |
| AT2G18960.1 | 1.49 | 0.56 | H(+)-ATPase 1 (HA1) |
| AT1G44542.1 | 1.87 | 0.55 | Cyclase family protein |
| AT5G38100.1 | 4.69 | 0.55 | S-adenosyl-L-methionine-dependent methyltransferases superfamily protein |
| AT2G44220.1 | 2.61 | 0.53 | Protein of Unknown Function (DUF239) |
| AT4G13770.1 | 1.57 | 0.52 | cytochrome P450, family 83, subfamily A, polypeptide 1 (CYP83A1) |
| AT2G43100.1 | 1.89 | 0.51 | isopropylmalate isomerase 2 (IPMI2) |
| AT5G23010.1 | 1.59 | 0.51 | methylthioalkylmalate synthase 1 (MAM1) |
| AT5G17330.1 | 1.94 | 0.50 | glutamate decarboxylase (GAD) |
| AT3G51920.1 | 2.14 | 0.50 | calmodulin 9 (CAM9) |
| AT4G23010.3 | 2.11 | 0.50 | UDP-galactose transporter 2 (UTR2) |
| AT3G53280.1 | 7.80 | 0.50 | cytochrome p450 71b5 (CYP71B5) |
| AT2G39200.1 | 1.80 | 0.49 | MILDEW RESISTANCE LOCUS O 12 (MLO12) |
| AT2G46572.1 | 39.72 | 0.49 | Potential natural antisense gene |
| AT4G10340.1 | 1.50 | 0.49 | light harvesting complex of photosystem II 5 (LHCB5) |
| AT3G19930.1 | 1.62 | 0.48 | sugar transporter 4 (STP4) |
| AT1G19715.3 | 1.53 | 0.48 | Mannose-binding lectin superfamily protein |
| AT5G40820.1 | 1.79 | 0.48 | Ataxia telangiectasia-mutated and RAD3-related (ATR) |
| AT5G67310.1 | 2.13 | 0.47 | cytochrome P450, family 81, subfamily G, polypeptide 1 (CYP81G1) |
| AT5G66690.1 | 1.72 | 0.47 | UGT72E2 |
| AT2G42690.1 | 2.28 | 0.47 | alpha/beta-Hydrolases superfamily protein |
| AT5G08640.2 | 4.09 | 0.47 | flavonol synthase 1 (FLS1) |
| AT5G64470.3 | 3.64 | 0.47 | INVOLVED IN: biological_process unknown |
| AT1G12110.1 | 1.57 | 0.46 | nitrate transporter 1.1 (NRT1.1) |
| AT1G48760.2 | 1.51 | 0.46 | delta-adaptin (delta-ADR) |
| AT1G21110.1 | 2.11 | 0.46 | O-methyltransferase family protein |
| Decreased expression | | | |
| AT4G36150.1 | 0.03 | -3.22 | Disease resistance protein (TIR-NBS-LRR class) family |
| AT4G33720.1 | 0.25 | -1.28 | CAP (Cysteine-rich secretory proteins, Antigen 5, and Pathogenesis-related 1 protein) superfamily protein |
| AT1G73260.1 | 0.43 | -1.15 | kunitz trypsin inhibitor 1 (KTI1) |
| AT5G18360.1 | 0.12 | -1.07 | Disease resistance protein (TIR-NBS-LRR class) family |
| AT3G47340.2 | 0.18 | -1.05 | glutamine-dependent asparagine synthase 1 (ASN1) |
| AT1G77520.1 | 0.34 | -1.04 | O-methyltransferase family protein |
| AT4G19690.2 | 0.43 | -0.96 | iron-regulated transporter 1 (IRT1) |
| AT5G54090.1 | 0.39 | -0.94 | DNA mismatch repair protein MutS, type 2 |
| AT2G25450.1 | 0.45 | -0.81 | 2-oxoglutarate (2OG) and Fe(II)-dependent oxygenase superfamily protein |
| AT1G21140.1 | 0.32 | -0.80 | Vacuolar iron transporter (VIT) family protein |
| AT4G21510.1 | 0.08 | -0.78 | F-box family protein |
| AT1G63220.1 | 0.11 | -0.76 | Calcium-dependent lipid-binding (CaLB domain) family protein |
| AT5G06730.1 | 0.38 | -0.74 | Peroxidase superfamily protein |
| AT4G01870.1 | 0.41 | -0.74 | tolB protein-related |
| AT3G21240.1 | 0.19 | -0.71 | 4-coumarate:CoA ligase 2 (4CL2) |
| AT1G78360.1 | 0.44 | -0.70 | glutathione S-transferase TAU 21 (GSTU21) |
| AT2G34210.1 | 0.00 | -0.70 | Transcription elongation factor Spt5 |
| AT5G05340.1 | 0.26 | -0.67 | Peroxidase superfamily protein |
| AT5G66170.3 | 0.32 | -0.61 | sulfurtransferase 18 (STR18) |
| AT2G37180.1 | 0.58 | -0.61 | RESPONSIVE TO DESICCATION 28 (RD28) |
| AT1G01580.1 | 0.42 | -0.59 | ferric reduction oxidase 2 (FRO2) |
| AT1G52810.1 | 0.21 | -0.58 | 2-oxoglutarate (2OG) and Fe(II)-dependent oxygenase superfamily protein |
| AT5G19440.1 | 0.58 | -0.58 | NAD(P)-binding Rossmann-fold superfamily protein |
| AT3G53260.1 | 0.65 | -0.54 | phenylalanine ammonia-lyase 2 (PAL2) |
| AT4G17030.1 | 0.28 | -0.54 | expansin-like B1 (EXLB1) |
| AT5G01595.1 | 0.36 | -0.51 | Potential natural antisense gene |
| AT1G50060.1 | 0.28 | -0.51 | CAP (Cysteine-rich secretory proteins, Antigen 5, and Pathogenesis-related 1 protein) superfamily protein |
| AT1G15040.2 | 0.33 | -0.50 | Class I glutamine amidotransferase-like superfamily protein |
| AT1G22410.1 | 0.61 | -0.50 | Class-II DAHP synthetase family protein |
| AT5G20620.1 | 0.60 | -0.50 | ubiquitin 4 (UBQ4) |
| AT1G75830.1 | 0.45 | -0.49 | low-molecular-weight cysteine-rich 67 (LCR67) |
| AT5G18370.1 | 0.40 | -0.49 | Disease resistance protein (TIR-NBS-LRR class) family |
| AT1G09780.1 | 0.62 | -0.48 | Phosphoglycerate mutase, 2,3-bisphosphoglycerate-independent |
| AT3G25510.1 | 0.38 | -0.48 | disease resistance protein (TIR-NBS-LRR class), putative |
| AT5G04950.1 | 0.48 | -0.47 | nicotianamine synthase 1 (NAS1) |
| AT3G21690.1 | 0.60 | -0.47 | MATE efflux family protein |
| AT2G41705.2 | 0.55 | -0.46 | camphor resistance CrcB family protein |
| AT3G61430.2 | 0.60 | -0.46 | plasma membrane intrinsic protein 1A (PIP1A) |
| AT4G12290.1 | 0.49 | -0.46 | Copper amine oxidase family protein |
| AT5G48930.1 | 0.55 | -0.46 | hydroxycinnamoyl-CoA shikimate/quinate hydroxycinnamoyl transferase (HCT) |

d. *S. pinnata* shoots

| ATID | fold difference | | effect | annotation |
| --- | --- | --- | --- | --- |
| Increased expression | | | | |
| AT1G59900.1 | | 2.58 | 1.18 | cold-regulated 47 (COR47) |
| AT1G68890.1 | | 3.97 | 1.13 | UDP-glucosyl transferase 85A2 (UGT85A2) |
| AT5G54390.1 | | 26.99 | 1.08 | DOX1 |
| AT4G15530.6 | | 2.56 | 1.05 | unknown protein |
| AT1G18590.1 | | 4.50 | 1.02 | glutamine-dependent asparagine synthase 1 (ASN1) |
| AT4G01800.2 | | 2.26 | 1.01 | glycine-rich protein 3 short isoform (GRP3S) |
| AT5G65220.1 | | 4.87 | 0.97 | cytochrome P450, family 81, subfamily G, polypeptide 1 (CYP81G1) |
| AT5G64040.1 | | 6.26 | 0.96 | kinectin-related |
| AT2G38120.1 | | 15.14 | 0.92 | K+ transporter 5 (KT5) |
| AT1G78860.1 | | 2.14 | 0.92 | Hyaluronan / mRNA binding family |
| AT5G61290.1 | | 2.16 | 0.90 | Ribosomal L29 family protein |
| AT1G78630.1 | | 2.69 | 0.90 | glycosylphosphatidylinositol-anchored lipid protein transfer 1 (LTPG1) |
| AT5G17890.1 | | 1.96 | 0.88 | NADH-dependent glutamate synthase 1 (GLT1) |
| AT1G08830.2 | | 2.01 | 0.85 | FUNCTIONS IN: molecular_function unknown |
| AT4G11310.1 | | 2.06 | 0.84 | GLYCINE RICH PROTEIN 7 (ATGRP7) |
| AT4G13430.1 | | 2.82 | 0.84 | Tudor/PWWP/MBT superfamily protein |
| AT5G09220.1 | | 2.14 | 0.83 | OBP3-responsive gene 1 (ORG1) |
| AT1G70890.1 | | 3.52 | 0.82 | UGT85A1 |
| AT2G21660.2 | | 2.08 | 0.80 | DEK domain-containing chromatin associated protein |
| AT2G28950.1 | | 1.95 | 0.78 | CP5 |
| AT2G38540.1 | | 2.06 | 0.78 | RNA-binding (RRM/RBD/RNP motifs) family protein |
| AT3G52930.1 | | 1.98 | 0.77 | photosystem II subunit R (PSBR) |
| AT4G32500.1 | | 2.03 | 0.77 | beta-xylosidase 1 (BXL1) |
| AT1G75750.2 | | 1.91 | 0.76 | pyruvate orthophosphate dikinase (PPDK) |
| AT1G22400.1 | | 15.48 | 0.75 | UDP-Glycosyltransferase superfamily protein |
| AT1G15810.1 | | 1.79 | 0.75 | S15/NS1, RNA-binding protein |
| AT4G26630.2 | | 2.24 | 0.74 | nitrile specifier protein 5 (NSP5) |
| AT1G27950.1 | | 2.42 | 0.71 | Tropomyosin-related |
| AT4G37800.1 | | 1.87 | 0.70 | LOW-TEMPERATURE-INDUCED 65 (LTI65) |
| AT1G35720.1 | | 2.33 | 0.70 | FUNCTIONS IN: molecular_function unknown |
| AT4G03050.1 | | 1.71 | 0.69 | aldehyde oxidase 1 (AAO1) |
| AT5G55660.1 | | 1.85 | 0.68 | PSAN |
| AT3G12780.1 | | 1.84 | 0.68 | GAST1 protein homolog 1 (GASA1) |
| AT3G02020.1 | | 1.84 | 0.67 | Pyridine nucleotide-disulphide oxidoreductase family protein |
| AT5G37770.1 | | 1.80 | 0.66 | myosin 2 (ATM2) |
| AT3G16470.3 | | 3.42 | 0.66 | nitrate reductase 1 (NIA1) |
| AT4G13615.1 | | 2.08 | 0.64 | unknown protein |
| AT1G03630.2 | | 2.31 | 0.64 | cytochrome P450, family 71, subfamily B, polypeptide 2 (CYP71B2) |
| AT2G31790.1 | | 3.23 | 0.63 | FUNCTIONS IN: molecular_function unknown |
| AT1G64720.1 | | 1.67 | 0.62 | eukaryotic translation initiation factor 3A (EIF3A) |
| AT5G46110.4 | | 2.24 | 0.62 | HARMLESS TO OZONE LAYER 1 (HOL1) |
| AT3G51950.2 | | 2.78 | 0.62 | D-mannose binding lectin protein with Apple-like carbohydrate-binding domain |
| AT5G48180.1 | | 2.19 | 0.62 | DEK domain-containing chromatin associated protein |
| AT1G58270.1 | | 48.85 | 0.62 | Papain family cysteine protease |
| AT2G43100.1 | | 3.60 | 0.62 | unknown protein |
| AT5G13630.2 | | 1.89 | 0.61 | ortholog of sugar beet HS1 PRO-1 2 (HSPRO2) |
| AT5G61790.1 | | 2.30 | 0.61 | pseudogene, similar to pathogen- and wound-inducible antifungal protein CBP20 precursor, similar to pathogen- and wound-inducible antifungal protein |
| Decreased expression | | | | |
| AT2G47180.1 | | 0.06 | -2.00 | Disease resistance protein (TIR-NBS-LRR class) family |
| AT1G52190.1 | | 0.28 | -1.64 | Papain family cysteine protease |
| AT4G13940.4 | | 0.21 | -1.59 | 2-oxoglutarate (2OG) and Fe(II)-dependent oxygenase superfamily protein |
| AT1G61520.1 | | 0.28 | -1.36 | extensin 3 (EXT3) |
| AT2G34210.1 | | 0.31 | -1.23 | methylthioalkylmalate synthase 1 (MAM1) |
| AT1G13080.2 | | 0.18 | -1.22 | branched-chain aminotransferase4 (BCAT4) |
| AT1G72290.1 | | 0.41 | -1.22 | cytochrome P450, family 83, subfamily A, polypeptide 1 (CYP83A1) |
| AT5G56000.1 | | 0.38 | -1.10 | methionine synthase 2 (MS2) |
| AT5G26000.1 | | 0.33 | -1.08 | isopropylmalate isomerase 2 (IPMI2) |
| AT1G21440.1 | | 0.46 | -1.05 | Class I glutamine amidotransferase-like superfamily protein |
| AT3G55700.1 | | 0.08 | -1.01 | Disease resistance protein (TIR-NBS-LRR class) family |
| AT3G47340.2 | | 0.25 | -1.00 | isopropylmalate dehydrogenase 1 |
| AT2G45960.3 | | 0.09 | -0.99 | Kunitz family trypsin and protease inhibitor protein |
| AT2G21660.2 | | 0.45 | -0.98 | beta glucosidase 18 (BGLU18) |
| AT2G26890.1 | | 0.38 | -0.96 | O-methyltransferase family protein |
| AT1G17745.2 | | 0.18 | -0.95 | cytochrome p450 79f1 (CYP79F1) |
| AT4G32260.1 | | 0.45 | -0.92 | phosphate 2 (PHO2) |
| AT3G03780.3 | | 0.50 | -0.92 | xyloglucan endotransglucosylase/hydrolase 7 (XTH7) |
| AT4G17520.1 | | 0.36 | -0.89 | unknown protein |
| AT5G53300.4 | | 0.23 | -0.84 | galactinol synthase 1 (GolS1) |
| AT4G26690.1 | | 0.41 | -0.82 | UDP-Glycosyltransferase superfamily protein |
| AT1G52000.1 | | 0.08 | -0.82 | AOP3 |
| AT5G64040.1 | | 0.31 | -0.82 | Auxin-Induced in Root cultures 12 (AIR12) |
| AT2G05380.2 | | 0.36 | -0.80 | Mannose-binding lectin superfamily protein |
| AT2G05520.6 | | 0.45 | -0.79 | Major facilitator superfamily protein |
| AT3G54600.1 | | 0.07 | -0.78 | DA1-related protein 4 (DAR4) |
| AT1G74090.1 | | 0.17 | -0.76 | ZW9 |
| AT4G05050.1 | | 0.36 | -0.76 | APS kinase (APK) |
| AT3G07390.1 | | 0.16 | -0.75 | polygalacturonase inhibiting protein 2 (PGIP2) |
| AT3G58610.3 | | 0.23 | -0.73 | NOD26-like intrinsic protein 6 |
| AT1G65980.2 | | 0.33 | -0.71 | Phosphoenolpyruvate carboxylase family protein |
| AT4G11320.1 | | 0.01 | -0.70 | TOUCH 2 (TCH2) |
| AT4G37800.1 | | 0.54 | -0.70 | PHE ammonia lyase 1 (PAL1) |
| AT2G32870.1 | | 0.36 | -0.69 | flavin-monooxygenase glucosinolate S-oxygenase 1 (FMO GS-OX1) |
| AT5G01220.1 | | 0.49 | -0.69 | AUXIN RESISTANT 1 (AUX1) |
| AT3G19710.1 | | 0.43 | -0.68 | amino acid permease 2 (AAP2) |
| AT1G51400.1 | | 0.46 | -0.68 | sulfotransferase 17 (SOT17) |
| AT1G77760.1 | | 0.24 | -0.67 | cellulose synthase-like A01 (CSLA01) |
| AT4G21990.1 | | 0.57 | -0.67 | glutathione S-transferase PHI 9 (GSTF9) |
| AT5G20960.2 | | 0.45 | -0.66 | DNA mismatch repair protein MutS, type 2 |
| AT5G53460.3 | | 0.48 | -0.66 | Flavin-binding monooxygenase family protein |
| AT5G52300.2 | | 0.49 | -0.66 | COBRA (COb) |
| AT5G54280.2 | | 0.47 | -0.66 | myb domain protein 28 (MYB28) |
| AT3G44300.1 | | 0.46 | -0.65 | glutathione S-transferase TAU 20 (GSTU20) |
| AT1G19920.1 | | 0.52 | -0.64 | Plant invertase/pectin methylesterase inhibitor superfamily |
| AT1G52400.3 | | 0.48 | -0.63 | APS2 |
| AT5G42530.1 | | 0.57 | -0.63 | HAL2-like (HL) |
| AT1G80760.1 | | 0.63 | -0.63 | PRXR1 |
| AT4G35160.1 | | 0.00 | -0.62 | Transcription elongation factor Spt5 |
| AT1G65860.1 | | 0.61 | -0.62 | isopropyl malate isomerase large subunit 1 (IIL1) |
| AT2G01140.1 | | 0.64 | -0.62 | glyceraldehyde-3-phosphate dehydrogenase B subunit (GAPb) |
| AT5G65220.1 | | 0.24 | -0.62 | pleiotropic drug resistance 1 (PDR1) |
| AT1G16410.1 | | 0.50 | -0.61 | Transmembrane amino acid transporter family protein |

**Table S2.** Top 100 significant (q-value < 0.005) differentially expressed genes between species. **(a)** roots with 0 µM Se, **(b)** roots with 20 µM Se, **(c)** shoots with 0 µM Se, and **(d)** shoots with 20 µM Se. Fold difference is calculated as the RPKM ratio of *S. pinnata*/*S. elata*. Effect refers to effect of magnitude. (+) or (-) values indicate more or less gene expression in *S. pinnata* relative to *S. elata*, respectively. The larger the absolute value of the effect is, the greater the treatment effect. Effect values were used to separate genes based on expression direction in descending order, with the most differentially expressed on top.

a. Root -Se

| ATID | fold difference | | effect | annotation |
| --- | --- | --- | --- | --- |
| More expressed | | | | |
| AT3G52930.1 | | 212.56 | 4.95 | Aldolase superfamily protein |
| AT1G19920.1 | | 111.72 | 4.68 | APS2 |
| AT3G16460.2 | | 221.44 | 4.44 | Mannose-binding lectin superfamily protein |
| AT2G45960.3 | | 61.89 | 4.23 | plasma membrane intrinsic protein 1B (PIP1b) |
| AT1G73260.1 | | 31.34 | 3.97 | kunitz trypsin inhibitor 1 (KTI1) |
| AT1G07890.8 | | 6E+13 | 3.95 | ascorbate peroxidase 1 (APX1) |
| AT4G36150.1 | | 225.37 | 3.84 | Disease resistance protein (TIR-NBS-LRR class) family |
| AT2G21045.1 | | 119.45 | 3.62 | Rhodanese/Cell cycle control phosphatase superfamily protein |
| AT2G01140.1 | | 41.77 | 3.58 | Aldolase superfamily protein |
| AT4G13940.4 | | 557.14 | 3.58 | HOMOLOGY-DEPENDENT GENE SILENCING 1 (HOG1) |
| AT5G62700.1 | | 2772.81 | 3.56 | tubulin beta chain 3 (TUB3) |
| AT5G40780.2 | | 507.39 | 3.37 | lysine histidine transporter 1 |
| AT4G13615.1 | | 460.08 | 3.31 | Uncharacterised protein family SERF |
| AT5G09810.1 | | 77.26 | 3.28 | actin 7 (ACT7) |
| AT3G23640.2 | | 74.07 | 3.28 | heteroglycan glucosidase 1 (HGL1) |
| AT4G16260.1 | | 158.57 | 3.26 | Glycosyl hydrolase superfamily protein |
| AT4G26690.1 | | 157.74 | 3.25 | SHAVEN 3 (SHV3) |
| AT2G05380.2 | | 6558.44 | 3.14 | glycine-rich protein 3 short isoform (GRP3S) |
| AT3G13330.1 | | 120.56 | 3.12 | proteasome activating protein 200 (PA200) |
| AT2G44790.1 | | 150.33 | 2.96 | uclacyanin 2 (UCC2) |
| AT4G33720.1 | | 1020.03 | 2.79 | CAP (Cysteine-rich secretory proteins, Antigen 5, and Pathogenesis-related 1 protein) superfamily protein |
| AT4G19690.2 | | 28.54 | 2.75 | iron-regulated transporter 1 (IRT1) |
| AT2G25490.1 | | 36.05 | 2.66 | EIN3-binding F box protein 1 (EBF1) |
| AT3G51950.2 | | 188.20 | 2.63 | Zinc finger (CCCH-type) family protein / RNA recognition motif (RRM)-containing protein |
| AT2G43610.1 | | 26.38 | 2.60 | Chitinase family protein |
| AT2G28780.1 | | 34.75 | 2.57 | unknown protein |
| AT3G28510.1 | | 228.65 | 2.56 | P-loop containing nucleoside triphosphate hydrolases superfamily protein |
| AT2G25450.1 | | 72.44 | 2.52 | 2-oxoglutarate (2OG) and Fe(II)-dependent oxygenase superfamily protein |
| AT3G52590.1 | | 18.94 | 2.52 | ubiquitin extension protein 1 (UBQ1) |
| AT4G38920.1 | | 73.31 | 2.50 | vacuolar-type H(+)-ATPase C3 (VHA-C3) |
| AT5G54090.1 | | 49.56 | 2.48 | DNA mismatch repair protein MutS, type 2 |
| AT1G52400.3 | | 8.56 | 2.46 | beta glucosidase 18 (BGLU18) |
| AT1G32450.1 | | 9.51 | 2.42 | nitrate transporter 1.5 (NRT1.5) |
| AT4G10340.1 | | 27.17 | 2.42 | light harvesting complex of photosystem II 5 (LHCB5) |
| AT3G26200.1 | | 58.23 | 2.40 | cytochrome P450, family 71, subfamily B, polypeptide 22 (CYP71B22) |
| AT2G46750.1 | | 16.58 | 2.38 | D-arabinono-1,4-lactone oxidase family protein |
| AT1G08830.2 | | 12.78 | 2.37 | copper/zinc superoxide dismutase 1 (CSD1) |
| AT5G54770.1 | | 3214.15 | 2.37 | THI1 |
| AT5G13490.2 | | 6.77 | 2.37 | ADP/ATP carrier 2 (AAC2) |
| AT5G37600.1 | | 15.73 | 2.35 | glutamine synthase clone R1 (GSR 1) |
| AT2G21660.2 | | 36.39 | 2.33 | GLYCINE RICH PROTEIN 7 (ATGRP7) |
| AT5G53300.4 | | 7.08 | 2.32 | ubiquitin-conjugating enzyme 10 (UBC10) |
| AT5G56630.1 | | 22.22 | 2.31 | phosphofructokinase 7 (PFK7) |
| AT4G21990.1 | | 95.84 | 2.30 | APS reductase 3 (APR3) |
| AT2G18960.1 | | 5.52 | 2.29 | H(+)-ATPase 1 (HA1) |
| AT1G19715.3 | | 41.31 | 2.28 | Mannose-binding lectin superfamily protein |
| AT4G15310.1 | | 5639.31 | 2.26 | cytochrome P450, family 702, subfamily A, polypeptide 3 (CYP702A3) |
| AT1G32790.2 | | 52.58 | 2.25 | CTC-interacting domain 11 (CID11) |
| AT1G15690.2 | | 6.51 | 2.25 | AVP1 |
| AT3G62830.2 | | 64.32 | 2.22 | UDP-GLUCURONIC ACID DECARBOXYLASE 2 (UXS2) |
| AT4G29040.1 | | 59.89 | 2.22 | regulatory particle AAA-ATPase 2A (RPT2a) |
| AT4G01290.2 | | 36.51 | 2.19 | unknown protein |
| AT3G02090.2 | | 66.24 | 2.16 | MPPBETA |
| AT5G64100.1 | | 31.59 | 2.16 | Peroxidase superfamily protein |
| AT4G33420.1 | | 28.72 | 2.16 | Peroxidase superfamily protein |
| AT5G19440.1 | | 16.44 | 2.15 | NAD(P)-binding Rossmann-fold superfamily protein |
| Less expressed | | | | |
| AT5G23020.1 | | 0.00 | -4.07 | 2-isopropylmalate synthase 2 (IMS2) |
| AT3G47470.1 | | 0.00 | -4.06 | light-harvesting chlorophyll-protein complex I subunit A4 (LHCA4) |
| AT3G16470.3 | | 0.04 | -3.86 | JASMONATE RESPONSIVE 1 (JR1) |
| AT5G13630.2 | | 0.00 | -3.71 | GENOMES UNCOUPLED 5 (GUN5) |
| AT1G70850.3 | | 0.06 | -3.45 | MLP-like protein 34 (MLP34) |
| AT1G68890.1 | | 0.01 | -3.19 | magnesium ion binding |
| AT4G11320.1 | | 0.00 | -3.18 | Papain family cysteine protease |
| AT2G26890.1 | | 0.01 | -2.98 | GRAVITROPISM DEFECTIVE 2 (GRV2) |
| AT1G45201.1 | | 0.02 | -2.91 | triacylglycerol lipase-like 1 (TLL1) |
| AT3G02020.1 | | 0.03 | -2.84 | aspartate kinase 3 (AK3) |
| AT5G52040.4 | | 0.02 | -2.82 | RNA-binding (RRM/RBD/RNP motifs) family protein |
| AT4G22100.1 | | 0.08 | -2.80 | beta glucosidase 2 (BGLU3) |
| AT1G78080.1 | | 0.00 | -2.74 | related to AP2 4 (RAP2.4) |
| AT3G03780.3 | | 0.12 | -2.69 | methionine synthase 2 (MS2) |
| AT3G26460.1 | | 0.00 | -2.68 | Polyketide cyclase/dehydrase and lipid transport superfamily protein |
| AT2G40130.2 | | 0.02 | -2.61 | Double Clp-N motif-containing P-loop nucleoside triphosphate hydrolases superfamily protein |
| AT1G52000.1 | | 0.09 | -2.60 | Mannose-binding lectin superfamily protein |
| AT3G63200.1 | | 0.03 | -2.59 | PATATIN-like protein 9 (PLP9) |
| AT2G38040.2 | | 0.05 | -2.57 | acetyl Co-enzyme a carboxylase carboxyltransferase alpha subunit (CAC3) |
| AT4G05050.1 | | 0.05 | -2.56 | ubiquitin 11 (UBQ11) |
| AT4G27640.1 | | 0.01 | -2.55 | ARM repeat superfamily protein |
| AT1G67090.2 | | 0.02 | -2.54 | ribulose bisphosphate carboxylase small chain 1A (RBCS1A) |
| AT1G65860.1 | | 0.04 | -2.51 | flavin-monooxygenase glucosinolate S-oxygenase 1 (FMO GS-OX1) |
| AT3G16640.1 | | 0.06 | -2.50 | translationally controlled tumor protein (TCTP) |
| AT3G02360.1 | | 0.04 | -2.46 | 6-phosphogluconate dehydrogenase family protein |
| AT1G09000.1 | | 0.02 | -2.43 | NPK1-related protein kinase 1 (NP1) |
| AT1G62770.1 | | 0.03 | -2.42 | Plant invertase/pectin methylesterase inhibitor superfamily protein |
| AT5G61790.1 | | 0.04 | -2.39 | calnexin 1 (CNX1) |
| AT4G39420.2 | | 0.04 | -2.35 | unknown protein |
| AT1G68750.1 | | 0.06 | -2.30 | phosphoenolpyruvate carboxylase 4 (PPC4) |
| AT3G19710.1 | | 0.09 | -2.30 | branched-chain aminotransferase4 (BCAT4) |
| AT3G54890.4 | | 0.05 | -2.30 | photosystem I light harvesting complex gene 1 (LHCA1) |
| AT1G48110.2 | | 0.05 | -2.29 | evolutionarily conserved C-terminal region 7 (ECT7) |
| AT1G15820.1 | | 0.03 | -2.28 | light harvesting complex photosystem II subunit 6 (LHCB6) |
| AT2G41840.1 | | 0.10 | -2.27 | Ribosomal protein S5 family protein |
| AT2G48130.1 | | 0.03 | -2.25 | Bifunctional inhibitor/lipid-transfer protein/seed storage 2S albumin superfamily protein |
| AT4G08150.1 | | 0.06 | -2.24 | KNOTTED-like from Arabidopsis thaliana (KNAT1) |
| AT5G44790.1 | | 0.05 | -2.23 | RESPONSIVE-TO-ANTAGONIST 1 (RAN1) |
| AT5G23010.1 | | 0.16 | -2.23 | methylthioalkylmalate synthase 1 (MAM1) |
| AT1G48920.1 | | 0.09 | -2.23 | nucleolin like 1 (NUC-L1) |
| AT3G04940.1 | | 0.03 | -2.22 | cysteine synthase D1 (CYSD1) |
| AT1G36160.2 | | 0.02 | -2.20 | acetyl-CoA carboxylase 1 (ACC1) |
| AT5G59950.5 | | 0.00 | -2.18 | RNA-binding (RRM/RBD/RNP motifs) family protein |
| AT5G23110.1 | | 0.01 | -2.16 | Zinc finger, C3HC4 type (RING finger) family protein |

a. Root +Se

| ATID | fold difference | | effect | annotation |
| --- | --- | --- | --- | --- |
| More expressed | | | | |
| AT1G19920.1 | 160.55 | 4.80 | | APS2 |
| AT3G52930.1 | 125.43 | 4.56 | | Aldolase superfamily protein |
| AT3G16460.2 | 702.61 | 4.36 | | Mannose-binding lectin superfamily protein |
| AT2G45960.3 | 73.74 | 4.08 | | plasma membrane intrinsic protein 1B (PIP1B) |
| AT2G21045.1 | 76.44 | 3.80 | | Rhodanese/Cell cycle control phosphatase superfamily protein |
| AT2G01140.1 | 82.18 | 3.70 | | Aldolase superfamily protein |
| AT5G62700.1 | 5E+13 | 3.61 | | tubulin beta chain 3 (TUB3) |
| AT5G40780.2 | 665.57 | 3.59 | | lysine histidine transporter 1 |
| AT2G18960.1 | 14.56 | 3.54 | | H(+)-ATPase 1 (HA1) |
| AT1G07890.8 | 5680.91 | 3.54 | | ascorbate peroxidase 1 (APX1) |
| AT3G23640.2 | 92.39 | 3.42 | | heteroglycan glucosidase 1 (HGL1) |
| AT4G13940.4 | 442.87 | 3.39 | | HOMOLOGY-DEPENDENT GENE SILENCING 1 (HOG1) |
| AT3G13330.1 | 191.27 | 3.30 | | proteasome activating protein 200 (PA200) |
| AT4G26690.1 | 87.77 | 3.30 | | SHAVEN 3 (SHV3) |
| AT4G13615.1 | 336.52 | 3.29 | | Uncharacterised protein family SERF |
| AT5G09810.1 | 54.24 | 3.26 | | actin 7 (ACT7) |
| AT4G16260.1 | 100.14 | 3.25 | | Glycosyl hydrolase superfamily protein |
| AT1G52400.3 | 25.56 | 3.25 | | beta glucosidase 18 (BGLU18) |
| AT1G73260.1 | 23.02 | 3.20 | | kunitz trypsin inhibitor 1 (KTI1) |
| AT5G54160.1 | 12.12 | 3.13 | | O-methyltransferase 1 (OMT1) |
| AT2G43610.1 | 84.04 | 3.11 | | Chitinase family protein |
| AT2G05380.2 | 1906.50 | 3.03 | | glycine-rich protein 3 short isoform (GRP3S) |
| AT1G15690.2 | 15.73 | 2.94 | | AVP1 |
| AT1G35720.1 | 11.14 | 2.86 | | annexin 1 (ANNAT1) |
| AT4G10340.1 | 33.41 | 2.83 | | light harvesting complex of photosystem II 5 (LHCB5) |
| AT2G44790.1 | 174.19 | 2.79 | | uclacyanin 2 (UCC2) |
| AT3G09260.1 | 8.43 | 2.78 | | PYK10 |
| AT1G21310.1 | 9.99 | 2.78 | | extensin 3 (EXT3) |
| AT2G25490.1 | 50.85 | 2.74 | | EIN3-binding F box protein 1 (EBF1) |
| AT5G54770.1 | 2E+13 | 2.73 | | THI1 |
| AT5G13490.2 | 10.29 | 2.71 | | ADP/ATP carrier 2 (AAC2) |
| AT3G51950.2 | 294.60 | 2.64 | | Zinc finger (CCCH-type) family protein / RNA recognition motif (RRM)-containing protein |
| AT1G19715.3 | 36.94 | 2.61 | | Mannose-binding lectin superfamily protein |
| AT5G37600.1 | 17.88 | 2.59 | | glutamine synthase clone R1 (GSR 1) |
| AT2G37040.1 | 8.65 | 2.49 | | PHE ammonia lyase 1 (PAL1) |
| AT3G12500.1 | 27.49 | 2.49 | | basic chitinase (HCHIB) |
| AT3G26200.1 | 35.26 | 2.41 | | cytochrome P450, family 71, subfamily B, polypeptide 22 (CYP71B22) |
| AT4G21990.1 | 120.75 | 2.40 | | APS reductase 3 (APR3) |
| AT4G38920.1 | 44.43 | 2.39 | | vacuolar-type H(+)-ATPase C3 (VHA-C3) |
| AT2G46750.1 | 11.64 | 2.38 | | D-arabinono-1,4-lactone oxidase family protein |
| AT1G32790.2 | 111.59 | 2.35 | | CTC-interacting domain 11 (CID11) |
| AT1G75220.1 | 182.66 | 2.31 | | Major facilitator superfamily protein |
| AT3G62830.2 | 62.93 | 2.30 | | UDP-GLUCURONIC ACID DECARBOXYLASE 2 (UXS2) |
| AT5G41670.2 | 26.51 | 2.29 | | 6-phosphogluconate dehydrogenase family protein |
| AT3G47730.1 | 12.53 | 2.29 | | ATP-binding cassette A2 (ABCA2) |
| AT1G59900.1 | 51.81 | 2.28 | | pyruvate dehydrogenase complex E1 alpha subunit (E1 ALPHA) |
| AT2G21660.2 | 26.29 | 2.27 | | GLYCINE RICH PROTEIN 7 (ATGRP7) |
| AT1G66580.1 | 9.12 | 2.25 | | senescence associated gene 24 (SAG24) |
| AT1G50010.1 | 6.08 | 2.23 | | tubulin alpha-2 chain (TUA2) |
| AT5G40510.1 | 28.39 | 2.23 | | Sucrase/ferredoxin-like family protein |
| AT4G15530.6 | 61.16 | 2.23 | | pyruvate orthophosphate dikinase (PPDK) |
| AT2G28780.1 | 23.28 | 2.23 | | unknown protein |
| AT1G32450.1 | 8.24 | 2.22 | | nitrate transporter 1.5 (NRT1.5) |
| AT2G15620.1 | 12.72 | 2.21 | | nitrite reductase 1 (NIR1) |
| AT3G28510.1 | 76.53 | 2.20 | | P-loop containing nucleoside triphosphate hydrolases superfamily protein |
| AT5G53300.4 | 6.18 | 2.20 | | ubiquitin-conjugating enzyme 10 (UBC10) |
| AT5G64100.1 | 20.75 | 2.17 | | Peroxidase superfamily protein |
| AT4G29010.1 | 47.35 | 2.13 | | ABNORMAL INFLORESCENCE MERISTEM (AIM1) |
| AT3G58610.3 | 8.89 | 2.13 | | ketol-acid reductoisomerase |
| AT1G20440.1 | 10.51 | 2.12 | | cold-regulated 47 (COR47) |
| Less expressed | | | | |
| AT3G41768.1 | 0.08 | -3.67 | | 18SrRNA |
| AT1G65860.1 | 0.02 | -3.42 | | flavin-monooxygenase glucosinolate S-oxygenase 1 (FMO GS-OX1) |
| AT5G13630.2 | 0.00 | -3.35 | | GENOMES UNCOUPLED 5 (GUN5) |
| AT1G68890.1 | 0.01 | -3.21 | | magnesium ion binding |
| AT3G47470.1 | 0.00 | -3.21 | | light-harvesting chlorophyll-protein complex I subunit A4 (LHCA4) |
| AT2G26890.1 | 0.01 | -3.14 | | GRAVITROPISM DEFECTIVE 2 (GRV2) |
| AT1G09000.1 | 0.01 | -3.13 | | NPK1-related protein kinase 1 (NP1) |
| AT1G78080.1 | 0.00 | -3.11 | | related to AP2 4 (RAP2.4) |
| AT1G45201.1 | 0.02 | -3.04 | | triacylglycerol lipase-like 1 (TLL1) |
| AT5G23110.1 | 0.01 | -2.92 | | Zinc finger, C3HC4 type (RING finger) family protein |
| AT4G22100.1 | 0.09 | -2.91 | | beta glucosidase 2 (BGLU3) |
| AT5G23020.1 | 0.01 | -2.90 | | 2-isopropylmalate synthase 2 (IMS2) |
| AT3G16640.1 | 0.05 | -2.84 | | translationally controlled tumor protein (TCTP) |
| AT5G52040.4 | 0.01 | -2.82 | | RNA-binding (RRM/RBD/RNP motifs) family protein |
| AT4G39420.2 | 0.03 | -2.82 | | unknown protein |
| AT3G16470.3 | 0.09 | -2.80 | | JASMONATE RESPONSIVE 1 (JR1) |
| AT3G02020.1 | 0.05 | -2.78 | | aspartate kinase 3 (AK3) |
| AT2G36420.1 | 0.04 | -2.78 | | unknown protein |
| AT1G70850.3 | 0.11 | -2.76 | | MLP-like protein 34 (MLP34) |
| AT5G49660.1 | 0.07 | -2.59 | | Leucine-rich repeat transmembrane protein kinase family protein |
| AT2G46950.1 | 0.04 | -2.54 | | cytochrome P450, family 709, subfamily B, polypeptide 2 (CYP709B2) |
| AT2G07709.1 | 0.04 | -2.53 | | pseudogene, similar to NADH dehydrogenase |
| AT4G02660.1 | 0.00 | -2.45 | | Beige/BEACH domain |
| AT3G63520.1 | 0.09 | -2.44 | | carotenoid cleavage dioxygenase 1 (CCD1) |
| AT5G02770.1 | 0.01 | -2.36 | | unknown protein |
| AT3G03780.3 | 0.15 | -2.35 | | methionine synthase 2 (MS2) |
| AT5G04380.1 | 0.03 | -2.35 | | S-adenosyl-L-methionine-dependent methyltransferases superfamily protein |
| AT4G01120.1 | 0.01 | -2.34 | | G-box binding factor 2 (GBF2) |
| AT3G28730.1 | 0.03 | -2.28 | | high mobility group (HMG) |
| AT3G22968.1 | 0.10 | -2.25 | | conserved peptide upstream open reading frame 59 (CPuORF59) |
| AT1G67090.2 | 0.04 | -2.23 | | ribulose bisphosphate carboxylase small chain 1A (RBCS1A) |
| AT1G15820.1 | 0.04 | -2.23 | | light harvesting complex photosystem II subunit 6 (LHCB6) |
| AT2G48130.1 | 0.03 | -2.21 | | Bifunctional inhibitor/lipid-transfer protein/seed storage 2S albumin superfamily protein |
| AT1G68750.1 | 0.07 | -2.21 | | phosphoenolpyruvate carboxylase 4 (PPC4) |
| AT5G49930.1 | 0.05 | -2.20 | | embryo defective 1441 (emb1441) |
| AT2G40130.2 | 0.04 | -2.18 | | Double Clp-N motif-containing P-loop nucleoside triphosphate hydrolases superfamily protein |
| AT1G36160.2 | 0.03 | -2.18 | | acetyl-CoA carboxylase 1 (ACC1) |
| AT4G08150.1 | 0.08 | -2.16 | | KNOTTED-like from Arabidopsis thaliana (KNAT1) |
| AT3G55460.1 | 0.06 | -2.16 | | SC35-like splicing factor 30 (SCL30) |
| AT3G56990.1 | 0.01 | -2.16 | | embryo sac development arrest 7 (EDA7) |

c. Shoot –Se

| ATID | fold difference | effect | annotation |  |
| --- | --- | --- | --- | --- |
| More expressed | | | |  |
| AT5G54770.1 | 2.44E+14 | 5.73 | THI1 |  |
| AT4G10340.1 | 84.26 | 5.68 | light harvesting complex of photosystem II 5 (LHCB5) |  |
| AT2G05100.1 | 10199.15 | 5.44 | photosystem II light harvesting complex gene 2.1 (LHCB2.1) |  |
| AT4G11310.1 | 78.24 | 4.22 | Papain family cysteine protease |  |
| AT2G45960.3 | 73.51 | 4.13 | plasma membrane intrinsic protein 1B (PIP1b) |  |
| AT1G60950.1 | 25.12 | 3.75 | FED A |  |
| AT1G07890.8 | 5E+13 | 3.67 | ascorbate peroxidase 1 (APX1) |  |
| AT5G62700.1 | 2288.39 | 3.63 | tubulin beta chain 3 (TUB3) |  |
| AT2G34410.2 | 149.25 | 3.50 | O-acetyltransferase family protein |  |
| AT5G09810.1 | 122.16 | 3.48 | actin 7 (ACT7) |  |
| AT1G73260.1 | 918.65 | 3.45 | kunitz trypsin inhibitor 1 (KTI1) |  |
| AT1G03630.2 | 107.21 | 3.43 | protochlorophyllide oxidoreductase C (POR C) |  |
| AT2G25450.1 | 660.28 | 3.28 | 2-oxoglutarate (2OG) and Fe(II)-dependent oxygenase superfamily protein |  |
| AT4G03280.2 | 18.76 | 3.25 | photosynthetic electron transfer C (PETC) |  |
| AT1G15810.1 | 74.17 | 3.19 | S15/NS1, RNA-binding protein |  |
| AT3G52930.1 | 99.82 | 3.10 | Aldolase superfamily protein |  |
| AT4G13615.1 | 314.24 | 3.05 | Uncharacterised protein family SERF |  |
| AT3G54600.1 | 11.04 | 3.04 | Class I glutamine amidotransferase-like superfamily protein |  |
| AT1G72600.2 | 13.09 | 3.00 | hydroxyproline-rich glycoprotein family protein |  |
| AT4G37800.1 | 14.28 | 2.99 | xyloglucan endotransglucosylase/hydrolase 7 (XTH7) |  |
| AT1G78630.1 | 24.98 | 2.98 | embryo defective 1473 (emb1473) |  |
| AT2G38540.1 | 15.02 | 2.96 | lipid transfer protein 1 (LP1) |  |
| AT1G74090.1 | 23.70 | 2.95 | desulfo-glucosinolate sulfotransferase 18 (SOT18) |  |
| AT3G51950.2 | 450.85 | 2.93 | Zinc finger (CCCH-type) family protein / RNA recognition motif (RRM)-containing protein |  |
| AT4G13940.4 | 532.49 | 2.92 | HOMOLOGY-DEPENDENT GENE SILENCING 1 (HOG1) |  |
| AT3G12780.1 | 31.37 | 2.89 | phosphoglycerate kinase 1 (PGK1) |  |
| AT4G21960.1 | 9.70 | 2.86 | PRXR1 |  |
| AT2G01140.1 | 43.74 | 2.86 | Aldolase superfamily protein |  |
| AT2G05380.2 | 108.12 | 2.84 | glycine-rich protein 3 short isoform (GRP3S) |  |
| AT4G38920.1 | 78.55 | 2.72 | vacuolar-type H(+)-ATPase C3 (VHA-C3) |  |
| AT4G26690.1 | 43.00 | 2.72 | SHAVEN 3 (SHV3) |  |
| AT2G46820.2 | 80.46 | 2.72 | photosystem I P subunit (PSI-P) |  |
| AT1G08830.2 | 18.67 | 2.70 | copper/zinc superoxide dismutase 1 (CSD1) |  |
| AT1G51400.1 | 88.96 | 2.69 | Photosystem II 5 kD protein |  |
| AT1G21310.1 | 30.12 | 2.68 | extensin 3 (EXT3) |  |
| AT1G42970.1 | 8.03 | 2.67 | glyceraldehyde-3-phosphate dehydrogenase B subunit (GAPb) |  |
| AT3G44300.1 | 2E+13 | 2.64 | nitrilase 2 (NIT2) |  |
| AT5G46110.4 | 7.90 | 2.64 | ACCLIMATION OF PHOTOSYNTHESIS TO  ENVIRONMENT 2 (APE2) |  |
| AT1G35720.1 | 9.13 | 2.63 | annexin 1 (ANNAT1) |  |
| AT2G25490.1 | 33.73 | 2.61 | EIN3-binding F box protein 1 (EBF1) |  |
| AT1G52400.3 | 12.78 | 2.60 | beta glucosidase 18 (BGLU18) |  |
| AT1G70830.4 | 32.31 | 2.60 | MLP-like protein 28 (MLP28) |  |
| AT4G28750.1 | 9.41 | 2.59 | PSA E1 KNOCKOUT (PSAE-1) |  |
| AT5G56000.1 | 9.28 | 2.57 | HEAT SHOCK PROTEIN 81.4 (Hsp81.4) |  |
| AT1G65980.2 | 102.19 | 2.55 | thioredoxin-dependent peroxidase 1 (TPX1) |  |
| AT1G20440.1 | 31.28 | 2.52 | cold-regulated 47 (COR47) |  |
| AT3G52590.1 | 23.43 | 2.52 | ubiquitin extension protein 1 (UBQ1) |  |
| AT1G70890.1 | 25.40 | 2.48 | MLP-like protein 43 (MLP43) |  |
| AT4G35160.1 | 115.74 | 2.43 | O-methyltransferase family protein |  |
| AT2G21660.2 | 63.30 | 2.42 | GLYCINE RICH PROTEIN 7 (ATGRP7) |  |
| AT4G21990.1 | 128.86 | 2.42 | APS reductase 3 (APR3) |  |
| AT2G30670.1 | 158.83 | 2.41 | NAD(P)-binding Rossmann-fold superfamily protein |  |
| AT2G05520.6 | 9.29 | 2.39 | glycine-rich protein 3 (GRP-3) |  |
| AT3G13330.1 | 43.92 | 2.38 | proteasome activating protein 200 (PA200) |  |
| AT2G28950.1 | 17.56 | 2.35 | expansin A6 (EXPA6) |  |
| AT2G26900.1 | 11.69 | 2.34 | Sodium Bile acid symporter family |  |
| AT4G36150.1 | 54.21 | 2.34 | Disease resistance protein (TIR-NBS-LRR class) family |  |
| AT1G17745.2 | 29.55 | 2.33 | D-3-phosphoglycerate dehydrogenase |  |
| AT2G13360.1 | 332.27 | 2.31 | alanine:glyoxylate aminotransferase (AGT) |  |
| AT3G58610.3 | 7.55 | 2.31 | ketol-acid reductoisomerase |  |
| AT1G59900.1 | 20.70 | 2.30 | pyruvate dehydrogenase complex E1 alpha subunit (E1 ALPHA) |  |
| AT5G54390.1 | 17.46 | 2.30 | HAL2-like (HL) |  |
| AT2G40100.1 | 117.81 | 2.30 | light harvesting complex photosystem II (LHCB4.3) |  |
| AT5G53300.4 | 7.11 | 2.28 | ubiquitin-conjugating enzyme 10 (UBC10) |  |
| Less expressed | | | |  |
| AT3G47470.1 | 0.00 | -6.69 | light-harvesting chlorophyll-protein complex I subunit A4 (LHCA4) |  |
| AT5G13630.2 | 0.00 | -5.25 | GENOMES UNCOUPLED 5 (GUN5) |  |
| AT4G35090.2 | 0.00 | -4.21 | catalase 2 (CAT2) |  |
| AT2G46950.1 | 0.01 | -4.02 | cytochrome P450, family 709, subfamily B, polypeptide 2 (CYP709B2) |  |
| AT4G32260.1 | 0.00 | -3.86 | ATPase, F0 complex, subunit B/B', bacterial/chloroplast |  |
| AT5G23020.1 | 0.01 | -3.75 | 2-isopropylmalate synthase 2 (IMS2) |  |
| AT1G67090.2 | 0.07 | -3.57 | ribulose bisphosphate carboxylase small chain 1A (RBCS1A) |  |
| AT1G68890.1 | 0.01 | -3.57 | magnesium ion binding |  |
| AT5G54270.1 | 0.00 | -3.53 | light-harvesting chlorophyll B-binding protein 3 (LHCB3) |  |
| AT1G15820.1 | 0.04 | -3.53 | light harvesting complex photosystem II subunit 6 (LHCB6) |  |
| AT1G61520.3 | 0.00 | -3.44 | photosystem I light harvesting complex gene 3 (LHCA3) |  |
| AT5G26000.1 | 0.01 | -3.40 | thioglucoside glucohydrolase 1 (TGG1) |  |
| AT4G11960.2 | 0.00 | -3.01 | PGR5-like B (PGRL1b) |  |
| AT3G23800.1 | 0.00 | -2.90 | selenium-binding protein 3 (SBP3) |  |
| AT5G52040.4 | 0.01 | -2.90 | RNA-binding (RRM/RBD/RNP motifs) family protein |  |
| AT3G04940.1 | 0.01 | -2.87 | cysteine synthase D1 (CYSD1) |  |
| AT1G61520.1 | 0.08 | -2.83 | photosystem I light harvesting complex gene 3 (LHCA3) |  |
| AT2G26890.1 | 0.01 | -2.80 | GRAVITROPISM DEFECTIVE 2 (GRV2) |  |
| AT1G52340.1 | 0.02 | -2.75 | ABA DEFICIENT 2 (ABA2) |  |
| AT2G32870.1 | 0.04 | -2.74 | TRAF-like family protein |  |
| AT3G59780.1 | 0.05 | -2.63 | Rhodanese/Cell cycle control phosphatase superfamily protein |  |
| AT4G22100.1 | 0.10 | -2.63 | beta glucosidase 2 (BGLU3) |  |
| AT3G54890.4 | 0.14 | -2.61 | photosystem I light harvesting complex gene 1 (LHCA1) |  |
| AT3G02020.1 | 0.04 | -2.59 | aspartate kinase 3 (AK3) |  |
| AT1G79040.1 | 0.13 | -2.56 | photosystem II subunit R (PSBR) |  |
| AT5G02020.2 | 0.02 | -2.52 | unknown protein |  |
| AT4G05050.1 | 0.07 | -2.50 | ubiquitin 11 (UBQ11) |  |
| AT5G58070.1 | 0.05 | -2.50 | temperature-induced lipocalin (TIL) |  |
| AT5G01220.1 | 0.08 | -2.46 | sulfoquinovosyldiacylglycerol 2 (SQD2) |  |
| AT5G40450.1 | 0.13 | -2.45 | unknown protein |  |
| AT3G16470.3 | 0.05 | -2.42 | JASMONATE RESPONSIVE 1 (JR1) |  |
| AT1G09000.1 | 0.02 | -2.39 | NPK1-related protein kinase 1 (NP1) |  |
| AT2G38040.2 | 0.08 | -2.37 | acetyl Co-enzyme a carboxylase carboxyltransferase alpha subunit (CAC3) |  |
| AT1G55480.1 | 0.02 | -2.35 | protein containing PDZ domain, a K-box domain, and a TPR region (ZKT) |  |
| AT5G61790.1 | 0.04 | -2.35 | calnexin 1 (CNX1) |  |
| AT4G01800.2 | 0.00 | -2.30 | Albino or Glassy Yellow 1 (AGY1) |  |

d. Shoot +Se

| ATID | fold difference | effect | annotation |
| --- | --- | --- | --- |
| More expressed | | | |
| AT2G05100.1 | 51346.62 | 5.96 | photosystem II light harvesting complex gene 2.1 (LHCB2.1) |
| AT5G54770.1 | 3E+14 | 5.90 | THI1 |
| AT4G10340.1 | 98.55 | 5.88 | light harvesting complex of photosystem II 5 (LHCB5) |
| AT2G45960.3 | 74.83 | 4.02 | plasma membrane intrinsic protein 1B (PIP1b) |
| AT1G15810.1 | 118.46 | 3.90 | S15/NS1, RNA-binding protein |
| AT2G05380.2 | 260.92 | 3.86 | glycine-rich protein 3 short isoform (GRP3S) |
| AT1G03630.2 | 169.98 | 3.80 | protochlorophyllide oxidoreductase C (POR C) |
| AT5G62700.1 | 5161.80 | 3.79 | tubulin beta chain 3 (TUB3) |
| AT2G38540.1 | 35.04 | 3.76 | lipid transfer protein 1 (LP1) |
| AT1G20440.1 | 75.89 | 3.68 | cold-regulated 47 (COR47) |
| AT2G34410.2 | 160.14 | 3.68 | O-acetyltransferase family protein |
| AT1G60950.1 | 21.81 | 3.60 | FED A |
| AT4G13615.1 | 522.75 | 3.59 | Uncharacterised protein family SERF |
| AT5G09810.1 | 137.20 | 3.55 | actin 7 (ACT7) |
| AT1G78630.1 | 34.33 | 3.39 | embryo defective 1473 (emb1473) |
| AT4G03280.2 | 20.33 | 3.33 | photosynthetic electron transfer C (PETC) |
| AT2G21660.2 | 148.40 | 3.29 | GLYCINE RICH PROTEIN 7 (ATGRP7) |
| AT1G07890.8 | 4E+14 | 3.24 | ascorbate peroxidase 1 (APX1) |
| AT2G05520.6 | 20.64 | 3.21 | glycine-rich protein 3 (GRP-3) |
| AT1G74090.1 | 24.36 | 3.10 | desulfo-glucosinolate sulfotransferase 18 (SOT18) |
| AT4G35160.1 | 242.86 | 3.02 | O-methyltransferase family protein |
| AT3G12780.1 | 34.00 | 2.98 | phosphoglycerate kinase 1 (PGK1) |
| AT1G51400.1 | 71.56 | 2.97 | Photosystem II 5 kD protein |
| AT4G28750.1 | 11.29 | 2.89 | PSA E1 KNOCKOUT (PSAE-1) |
| AT5G56000.1 | 12.25 | 2.87 | HEAT SHOCK PROTEIN 81.4 (Hsp81.4) |
| AT3G52590.1 | 35.57 | 2.87 | ubiquitin extension protein 1 (UBQ1) |
| AT3G52930.1 | 89.02 | 2.86 | Aldolase superfamily protein |
| AT1G75750.2 | 79.58 | 2.80 | GAST1 protein homolog 1 (GASA1) |
| AT1G72600.2 | 12.19 | 2.80 | hydroxyproline-rich glycoprotein family protein |
| AT4G11310.1 | 38.14 | 2.80 | Papain family cysteine protease |
| AT4G13940.4 | 502.80 | 2.79 | HOMOLOGY-DEPENDENT GENE SILENCING 1 (HOG1) |
| AT4G21990.1 | 207.58 | 2.78 | APS reductase 3 (APR3) |
| AT1G73260.1 | 93.49 | 2.72 | kunitz trypsin inhibitor 1 (KTI1) |
| AT2G30670.1 | 221.07 | 2.70 | NAD(P)-binding Rossmann-fold superfamily protein |
| AT3G51950.2 | 238.95 | 2.64 | Zinc finger (CCCH-type) family protein / RNA recognition motif (RRM)-containing protein |
| AT2G01140.1 | 34.30 | 2.63 | Aldolase superfamily protein |
| AT1G75350.1 | 388.13 | 2.56 | embryo defective 2184 (emb2184) |
| AT1G35720.1 | 8.15 | 2.56 | annexin 1 (ANNAT1) |
| AT5G53300.4 | 8.85 | 2.51 | ubiquitin-conjugating enzyme 10 (UBC10) |
| AT1G70890.1 | 38.34 | 2.49 | MLP-like protein 43 (MLP43) |
| AT1G08830.2 | 19.14 | 2.48 | copper/zinc superoxide dismutase 1 (CSD1) |
| AT4G34350.1 | 9.32 | 2.46 | 4-hydroxy-3-methylbut-2-enyl diphosphate reductase (HDR) |
| AT1G15340.2 | 278.76 | 2.46 | methyl-CPG-binding domain 10 (MBD10) |
| AT2G13360.1 | 268.41 | 2.42 | alanine:glyoxylate aminotransferase (AGT) |
| AT1G20693.3 | 9.24 | 2.42 | high mobility group B2 (HMGB2) |
| AT1G59900.1 | 21.70 | 2.40 | pyruvate dehydrogenase complex E1 alpha subunit (E1 ALPHA) |
| AT1G70830.4 | 28.20 | 2.39 | MLP-like protein 28 (MLP28) |
| AT1G17745.2 | 28.21 | 2.35 | D-3-phosphoglycerate dehydrogenase |
| AT5G46110.4 | 6.22 | 2.31 | ACCLIMATION OF PHOTOSYNTHESIS TO  ENVIRONMENT 2 (APE2) |
| AT2G26900.1 | 12.49 | 2.30 | Sodium Bile acid symporter family |
| AT3G58610.3 | 7.19 | 2.30 | ketol-acid reductoisomerase |
| AT5G53450.2 | 15.59 | 2.29 | OBP3-responsive gene 1 (ORG1) |
| AT4G38920.1 | 66.02 | 2.29 | vacuolar-type H(+)-ATPase C3 (VHA-C3) |
| AT4G37800.1 | 9.14 | 2.27 | xyloglucan endotransglucosylase/hydrolase 7 (XTH7) |
| AT5G52300.2 | 13.41 | 2.23 | LOW-TEMPERATURE-INDUCED 65 (LTI65) |
| Less expressed | | | |
| AT3G47470.1 | 0.00 | -7.05 | light-harvesting chlorophyll-protein complex I subunit A4 (LHCA4) |
| AT5G13630.2 | 0.00 | -5.84 | GENOMES UNCOUPLED 5 (GUN5) |
| AT4G35090.2 | 0.00 | -4.34 | catalase 2 (CAT2) |
| AT2G46950.1 | 0.01 | -4.20 | cytochrome P450, family 709, subfamily B, polypeptide 2 (CYP709B2) |
| AT4G32260.1 | 0.00 | -3.76 | ATPase, F0 complex, subunit B/B', bacterial/chloroplast |
| AT5G54270.1 | 0.00 | -3.76 | light-harvesting chlorophyll B-binding protein 3 (LHCB3) |
| AT1G67090.2 | 0.06 | -3.75 | ribulose bisphosphate carboxylase small chain 1A (RBCS1A) |
| AT5G23020.1 | 0.00 | -3.74 | 2-isopropylmalate synthase 2 (IMS2) |
| AT1G61520.3 | 0.00 | -3.56 | photosystem I light harvesting complex gene 3 (LHCA3) |
| AT1G15820.1 | 0.06 | -3.34 | light harvesting complex photosystem II subunit 6 (LHCB6) |
| AT1G68890.1 | 0.02 | -3.13 | magnesium ion binding |
| AT1G61520.1 | 0.06 | -3.06 | photosystem I light harvesting complex gene 3 (LHCA3) |
| AT4G11960.2 | 0.00 | -3.04 | PGR5-like B (PGRL1b) |
| AT2G26890.1 | 0.01 | -2.99 | GRAVITROPISM DEFECTIVE 2 (GRV2) |
| AT1G62380.1 | 0.08 | -2.89 | ACC oxidase 2 (ACO2) |
| AT3G02020.1 | 0.01 | -2.89 | aspartate kinase 3 (AK3) |
| AT3G03780.3 | 0.10 | -2.85 | methionine synthase 2 (MS2) |
| AT4G05050.1 | 0.04 | -2.83 | ubiquitin 11 (UBQ11) |
| AT1G52340.1 | 0.02 | -2.82 | ABA DEFICIENT 2 (ABA2) |
| AT3G59780.1 | 0.03 | -2.72 | Rhodanese/Cell cycle control phosphatase superfamily protein |
| AT5G52040.4 | 0.02 | -2.66 | RNA-binding (RRM/RBD/RNP motifs) family protein |
| AT3G04940.1 | 0.01 | -2.65 | cysteine synthase D1 (CYSD1) |
| AT5G26000.1 | 0.02 | -2.61 | thioglucoside glucohydrolase 1 (TGG1) |
| AT1G54780.1 | 0.00 | -2.56 | TLP18.3 |
| AT4G03050.1 | 0.01 | -2.55 | AOP3 |
| AT1G01790.1 | 0.02 | -2.53 | K+ efflux antiporter 1 (KEA1) |
| AT2G32870.1 | 0.06 | -2.49 | TRAF-like family protein |
| AT4G19840.1 | 0.08 | -2.47 | phloem protein 2-A1 (PP2-A1) |
| AT3G19710.1 | 0.06 | -2.39 | branched-chain aminotransferase4 (BCAT4) |
| AT1G55480.1 | 0.02 | -2.36 | protein containing PDZ domain, a K-box domain, and a TPR region (ZKT) |
| AT1G09000.1 | 0.02 | -2.35 | NPK1-related protein kinase 1 (NP1) |
| AT1G16410.1 | 0.04 | -2.34 | cytochrome p450 79f1 (CYP79F1) |
| AT5G02020.2 | 0.03 | -2.32 | unknown protein |
| AT4G22100.1 | 0.13 | -2.32 | beta glucosidase 2 (BGLU3) |
| AT5G58070.1 | 0.07 | -2.32 | temperature-induced lipocalin (TIL) |
| AT3G23800.1 | 0.00 | -2.31 | selenium-binding protein 3 (SBP3) |
| AT5G23010.1 | 0.13 | -2.30 | methylthioalkylmalate synthase 1 (MAM1) |
| AT3G25920.1 | 0.05 | -2.30 | ribosomal protein L15 (RPL15) |
| AT5G49660.1 | 0.05 | -2.28 | Leucine-rich repeat transmembrane protein kinase family protein |
| AT4G01800.2 | 0.00 | -2.26 | Albino or Glassy Yellow 1 (AGY1) |
| AT5G01220.1 | 0.11 | -2.26 | sulfoquinovosyldiacylglycerol 2 (SQD2) |
| AT3G63520.1 | 0.13 | -2.26 | carotenoid cleavage dioxygenase 1 (CCD1) |
| AT4G39420.2 | 0.04 | -2.26 | unknown protein |
| AT2G38040.2 | 0.11 | -2.25 | acetyl Co-enzyme a carboxylase carboxyltransferase alpha subunit (CAC3) |
| AT5G61790.1 | 0.05 | -2.25 | calnexin 1 (CNX1) |

**Table S3.** List of primers used in qRT-PCR reactions.

| **Gene** | **Forward (5’- 3’)** | **Reverse (5’- 3’)** |
| --- | --- | --- |
| *sultr1;2* | TAGTGATTGCTGCGAGGATG | CGTCGTTCTCTTGACATTGC |
| *aps1* | CCCTATCCTTTTGCTTCATCC | GTGCTGCTTCATCCTCCAAC |
| *aps2* | CATCAAGAGGAACATCATCAGC | TTACAGGCTATCTCCAAAACAGC |
| *act1* | AGCATGAAGATCAAGGTGGTG | CTGACTCATCGTACTCTCCCT |
